# Glutathione S-transferase: a candidate gene for berry color in muscadine grapes (*Vitis rotundifolia*)

**DOI:** 10.1093/g3journal/jkac060

**Published:** 2022-03-18

**Authors:** Aruna Varanasi, Margaret Worthington, Lacy Nelson, Autumn Brown, Thomas Mason Chizk, Renee Threlfall, Luke Howard, Patrick Conner, Rosa Figueroa-Balderas, Mélanie Massonnet, Dario Cantu, John R Clark

**Affiliations:** 1 Department of Horticulture, University of Arkansas, Fayetteville, AR 72701, USA; 2 Department of Food Science, University of Arkansas, Fayetteville, AR 72701, USA; 3 Department of Horticulture, University of Georgia, Tifton, GA 31793, USA; 4 Department of Viticulture & Enology, University of California, Davis, Davis, CA 95616, USA

**Keywords:** glutathione S-transferase, anthocyanin, *Vitis*, berry color, *Muscadinia rotundifolia*

## Abstract

Muscadine grapes (*Vitis rotundifolia* Michx.) are a specialty crop cultivated in the southern United States. Muscadines (2*n* = 40) belong to the *Muscadinia* subgenus of *Vitis*, while other cultivated grape species belong to the subgenus *Euvitis* (2*n* = 38). The muscadine berry color locus was mapped to a 0.8 Mbp region syntenic with chromosome 4 of *Vitis vinifera*. In this study, we identified glutathione S-transferase4 as a likely candidate gene for anthocyanin transport within the berry color locus. PCR and Kompetitive allele-specific PCR genotyping identified a single intragenic SNP (C/T) marker corresponding to a proline to leucine mutation within the muscadine glutathione S-transferase4 (*VrGST4*) that differentiated black (CC and CT) from bronze (TT) muscadines in 126 breeding selections, 76 cultivars, and 359 progeny from 3 mapping populations. Anthocyanin profiling on a subset of the progeny indicated a dominant *VrGST4* action. *VrGST4* was expressed in skins of both black and bronze muscadines at similar levels. While nonsynonymous polymorphisms between black and bronze muscadines were discovered in *VrGSTF12*, another Type I GST-coding gene in the muscadine color locus, this gene was ruled out as a possible candidate for berry color because RNA sequencing indicated it is not expressed in berry skins at véraison from black or bronze genotypes. These results suggest that the bronze phenotype in muscadines is regulated by a mechanism distinct from the *MybA* gene cluster responsible for berry color variation in *Vitis vinifera*.

## Introduction

Muscadine grapes (*Vitis rotundifolia* Michx., syn. *Muscadinia rotundifolia*) are a specialty fruit crop native to the southeastern United States. Wild and cultivated muscadine grapes are commonly found from Delaware to central Florida and from the Atlantic coast to eastern Texas (Lane 1997). The genus *Vitis* is divided into 2 subgenera, *Euvitis* Planch. (bunch grapes) and *Muscadinia* Planch. All bunch grape species, such as the European wine and table grape (*Vitisvinifera*) and the American “Concord” grape (*V. labrusca*), belong to *Euvitis*, whereas *Muscadinia* consists of only 3 species, *V. munsoniana* Simpson ex Munson, *V. popenoei* Fennell, and muscadine grapes (Wen 2007). Of the 3 species in subgenus *Muscadinia*, only muscadine grapes have commercial value (Brizicky 1965; Olien 1990). Muscadine grapes are tolerant to several pests and diseases such as grape phylloxera (*Daktulosphaira vitifoliae* Fitch) (Firoozabady and Olmo 1982), Pierce’s disease (*Xylella fastidiosa*) (Olmo 1971), and other major fungal pathogens (Staudt 1997; Merdinoglu *et al.* 2003) that cause extensive losses in *V. vinifera*.

Muscadine grapes possess distinct genetic and morphological characteristics that differentiate them from bunch grape species in subgenus *Euvitis*. Muscadine vines have unbranched tendrils and produce smaller clusters of berries that shatter at maturity. Berries are larger with thick skins and large bitter seeds (Striegler *et al.* 2005; Morris and Brady 2007). They also possess a unique foxy and candy-like aroma distinct from bunch grape species (Baek *et al.* 1997). Muscadines and other *Muscadinia* grapes (2*x* = 2*n* = 40) also differ in the number of somatic chromosomes from bunch grapes (2*x* = 2*n* = 38). They are considered a source of genetic variation and disease resistance for the *Euvitis* species but are not commonly used for hybridization with bunch grapes due to the difference in the chromosomes (Olmo 1986) and graft-incompatibility (Olien 1990). However, muscadine cultivation has gained importance in recent years for fresh fruit, wine, and juice production. Furthermore, the dried pomace (left over fruit after wine and juice processing) is being used as a functional food due to its high nutraceutical content (Vashisth *et al.* 2011).

Muscadine breeding is complex since it requires the simultaneous selection of several important traits. The most common challenge for muscadine breeding programs is to increase fruit quality while retaining disease resistance and vine vigor. Breeding efforts are currently focused on selection for seedlessness, hermaphroditism, and stable berry color during processing (Lu *et al.* 1993; Conner and MacLean 2013; Xu *et al.* 2014; Conner *et al.* 2017; Lewter *et al.* 2019). Berry color of muscadine grapes is especially important for the wine and juice industry as poor color stability can result in reduced quality of processed products (Morris and Brady 2007; Vasanthaiah *et al.* 2011). While muscadines cover a spectrum of berry shades, there are 2 primary color types; black (very dark purple) or bronze (greenish-yellow) (Conner and MacLean 2013). Black-fruited muscadines are much more common in the wild, though bronze muscadines are also occasionally found, and bronze berry color is recessively inherited (Stuckey 1919). Both black and bronze muscadine cultivars are available for the fresh market and processing industries.

The color of muscadine berries is determined by the quantity and composition of anthocyanins, which are accumulated in the skins. Quality and color stability of muscadine juice and wine are affected by the total anthocyanin content, anthocyanin composition, and intra-molecular copigmentation (Sims and Bates 1994; Talcott *et al.* 2003). Six types of anthocyanins (malvidin, peonidin, pelargonidin, petunidin, cyanidin, and delphinidin) have been identified in *V. vinifera* and muscadine grapes (He *et al.* 2010; Conner and MacLean 2013). While the anthocyanins in bunch grape species are acylated 3-O-monoglucosides, among which malvidin is more common (Jánváry *et al.* 2009), muscadine grapes have nonacylated 3,5-O-diglucosidic anthocyanins with delphinidin being the predominant type (Ballinger *et al.* 1974; Sandhu and Gu 2010). Conner and MacLean (2013) examined anthocyanin content and composition from the berry skin extracts of 22 *V. rotundifolia* cultivars and *Muscadinia* germplasm and found that anthocyanin content ranged from less than 100 µg.g^−1^ in bronze muscadines to over 5,000 µg.g^−1^ in the highly pigmented black muscadines.

Anthocyanins are synthesized by the flavonoid biosynthetic pathway, which is one of the best studied pathways in plants (Tanaka *et al.* 2008; He *et al.* 2010). Anthocyanin biosynthesis in plants occurs within the cytosol and the end products are transported to be deposited in the vacuoles. Regulation of genes involved in anthocyanin biosynthesis has been studied in several plant species (Cone *et al.* 1986; Quattrocchio *et al.* 1993; Holton and Cornish 1995; Zhao *et al.* 2015). While the coordinated expression of different structural genes appears to be regulated by the MYC and MYB transcription factors (Kobayashi *et al.* 2002; Deluc *et al.* 2006), their transport to the final site of storage, which is a critical process for pigmentation, is facilitated by several mechanisms including glutathione S-transferase (GST)-mediated transport (Dixon *et al.* 2010). GSTs comprise a large multigenic family in plants with diverse functions in the cell. They are soluble membrane-associated dimers known to function enzymatically in cellular detoxification through conjugation of xenobiotic substrates with glutathione (GSH) (Dixon and Edwards 2005). In addition, GSTs function in a noncatalytic role by acting as carrier proteins (ligandins) for shuttling of several endogenous compounds including vacuolar sequestration of anthocyanins (Dixon *et al.* 2010). GSTs are known to play a major role in anthocyanin transport in several plants such as *ZmBz2* from maize (*Zea mays*) (Marrs *et al.* 1995), *PhAn9* from petunia (*Petunia hybrida*) (Mueller *et al.* 2000), *AtTT19* from *Arabidopsis thaliana* (Sun *et al.* 2012), and *VvGST4* from *V. vinifera* (Conn *et al.* 2008; Pérez-Díaz *et al.* 2016).

Genes controlling berry color have been well characterized in *V. vinifera*. The color locus in *V. vinifera* was mapped to a 5 Mbp region on chromosome 2 and was associated with a single *MybA* gene cluster that accounted for 84% of the color variation (Fournier-Level *et al.* 2009; Myles *et al.* 2011). This variation in berry color is the result of additive effects from alleles of 3 MYB-type transcription factor genes, *VvMybA1*, *VvMybA2*, and *VvMybA3*, within the single *MybA* gene cluster. While *VvMybA1* and *VvMybA2* were demonstrated to be functionally involved in berry pigmentation (Kobayashi *et al.* 2002; Walker *et al.* 2007), the association of *VvMybA3* was determined purely through statistics-based approach due to the strong LD detected with *VvMybA2* (Fournier-Level *et al.* 2009). Five polymorphisms (1 retrotransposon, 3 SNPs, and one 2-bp In/Del) within these *MybA* genes caused structural changes in the *MybA* promoters and MYB proteins that resulted in the quantitative variation of berry color in *V. vinifera* (This *et al.* 2007; Fournier-Level *et al.* 2009). White color of *V. vinifera* berries is inherited as a recessive trait and has been linked to the presence of a single gypsy-type retrotransposon, *Gret1*, in homozygous condition in the promoter of *VvMybA1* (Kobayashi *et al.* 2004; This *et al.* 2007; Fournier-Level *et al.* 2009). Although the *Gret1* transposon in *VvMybA1* is a major determinant for generating color variation, the 3 SNPs detected in the exon of *VvMybA1* and the single In/Del identified in the exon of *VvMybA2* also contributed significantly (23% of the variance) to the quantitative variation of anthocyanin content in *V. vinifera* (Fournier-Level *et al.* 2009).

So far, limited research has been conducted on the genetic control of berry color and its association with anthocyanin content in *V. rotundifolia*. Technological advances in *V. vinifera* genomics (Venturini *et al.* 2013; Fennell *et al.* 2015; Chin *et al.* 2016; Yang *et al.* 2016) and the recent development of 2 new chromosome-scale reference assemblies of the muscadine cultivars “Trayshed” and “Noble” (Cochetel *et al.* 2021; Park *et al.* 2022) have enabled the application of genomic resources and tools from *V. vinifera* to advance molecular genetic analysis in *V. rotundifolia* (Lewter *et al.* 2019). Using 2 biparental F1 mapping populations segregating for flower sex and berry color with “Black Beauty” or “Supreme” being the female parent and “Nesbitt” as the common male parent, Lewter *et al.* (2019) developed the first saturated genotyping by sequencing-based linkage maps in muscadine grape. All 3 parents were heterozygous for the black phenotype and the progeny in both populations segregated at a 3:1 ratio for black to bronze berry color. The dense linkage maps were each composed of 20 linkage groups (LGs), with over 1,200 markers in the “Black Beauty” × “Nesbitt” population and over 2,000 markers in “Supreme” × “Nesbitt” population. A high degree of colinearity was observed between these genetic maps and the physical map of *V. vinifera* except for LGs 7 and 20, suggesting a highly conserved genome structure between the 2 species. The markers from LGs 7 and 20 of *V. rotundifolia* colocalized to chromosome 7 in *V. vinifera* suggesting a possible split of chromosome 7 in *V. vinifera* or a fusion of the 2 chromosomes from *V. rotundifolia* during *Vitis* evolution (Lewter *et al.* 2019).

The berry color locus mapped to a region corresponding to 11.1–11.9 Mbp on chromosome 4 on the physical map of *V. vinifera* in both muscadine populations (Lewter *et al.* 2019). This is an interesting finding, as the berry color locus in *V. vinifera* was mapped to chromosome 2 where *MybA* genes were identified as candidate genes (Fournier-Level *et al.* 2009) and suggests that other genes in the anthocyanin biosynthesis pathway possibly determine berry color in *V. rotundifolia*. Therefore, the objective of this study was to identify potential candidate genes within the 0.8 Mbp mapped locus on chromosome 4 of *V. vinifera* and validate their association with berry color variation in *V. rotundifolia*.

## Materials and methods

### Plant material

Leaf tissue and berry skins from 4 muscadine cultivars used as parents or grandparents for the 2 biparental F_1_ mapping populations (“Black Beauty” × “Nesbitt” and “Supreme” × “Nesbitt”) developed for mapping the color locus (Lewter *et al.* 2019) were initially used in this study to search for potential candidate genes. While the 3 parents, “Black Beauty,” “Supreme,” and “Nesbitt,” are heterozygous for black berry color, “Fry” is the recessive bronze-fruited cultivar found prominently in the pedigree of all 3 black-fruited parents (Goldy and Nesbitt 1985; Clark 1997; Conner 2013; Lewter *et al.* 2019). Berry skins at véraison were collected from 3 additional black-fruited genotypes (AM-70, NC67A015_26, and “Noble”) and 3 bronze-fruited genotypes (“Carlos,” “Fry,” and “Summit”). Plant materials (young leaves and berries) for many of the muscadine samples used in this study were collected from the University of Arkansas System Division of Agriculture (UA) Fruit Research Station (FRS) in Clarksville, AR. Additional leaf samples used for Kompetitive allele-specific PCR (KASP) genotyping were provided by the University of Georgia (UGA; Tifton, GA), the University of California Davis (UCD), a private research farm maintained by Jeff Bloodworth in Hillsborough, NC, and the USDA National Clonal Germplasm Repository (NCGR; Davis, CA).

### DNA extraction, Total RNA isolation, and cDNA synthesis

DNA was extracted from young leaves using a modified CTAB procedure (Porebski *et al*. 1997). Berry skins at véraison were collected on dry ice and stored at −80°C until RNA isolation. Total RNA was isolated from the frozen berry skins using Spectrum plant total RNA kit (Sigma-Aldrich, St. Louis, MO, USA) according to the manufacturer’s instructions. Integrity of the total RNA isolated was confirmed by agarose gel electrophoresis and the quantity and purity of double-stranded DNA and RNA was verified using a Qubit Fluorometer (Thermo Fisher Scientific, Waltham, MA, USA). First strand cDNA was synthesized from 500 ng of total RNA from each sample using iScript cDNA synthesis kit (Bio-Rad Laboratories Inc., Hercules, CA, USA) following manufacturer’s instructions.

### Identification of a candidate gene in the *V. vinifera* and *V. rotundifolia* reference genomes

The annotated genes within the 11.1–11.9 Mbp interval on chromosome 4 of the 12X.0 version of the PN40024 (NCBI accession NC_012010.3) *V. vinifera* reference genome were explored to identify genes associated with anthocyanin biosynthesis and compared with known sequences in the NCBI database using BLAST. Two genes annotated as GST in this region were explored as potential candidates: *VvGST4* (ID V0: GSVIVG01035256001; ID V1: VIT_04s0079g00690) and another a phi-type GST-coding gene (ID V0: GSVIVG01035262001; ID V1: VIT_04s0079g00710). Homologs of *VvGST4* (*VrGST4*, VITMroTrayshed_v2.0.hap1.chr04.ver2.0.g046320) and the phi-type GST-coding gene (*VrGSTF12*, VITMroTrayshed_v2.0.hap1.chr04.ver2.0.g046340) were subsequently identified in the “Trayshed” (Cochetel *et al.* 2021) and “Noble” (Park *et al.* 2022) genome assemblies.

Raw genomic DNA sequencing data of “Regale” muscadine (NCBI BioSamples SAMN07446122 and SAMN07446115) were trimmed for quality and length using Trimmomatic (Bolger *et al.* 2014) and aligned to the “Trayshed” assembly with BWA MEM (Li 2013) for purposes of additional sequence comparison. “Regale” alignments were deduplicated, formatted, and called for variants using SAMtools and BCFtools (Danecek *et al.* 2021).

### PCR amplification and sequencing

The glutathione S-transferase4 (*GST4*) candidate gene from “Black Beauty,” “Supreme,” “Nesbitt,” and “Fry” was amplified using primers designed from the *VvGST4* sequence. For initial PCR, genomic DNA was used to amplify the *GST4* sequence using forward primer 5′-ATATCAAGCAGCGAGCTCCA-3′ and reverse primer 5′-CCTCTTGGGAAAAAGCTTGG-3′. To isolate the full-length *GST4* sequence, forward primer 5′-ATATCAAGCAGCGAGCTCCA-3′ and reverse primer 5′-GGTGGAAGATGGTGATGAAGGT-3′ were used on cDNA.

We attempted to isolate the full-length *VrGSTF12* sequences of “Black Beauty,” “Supreme,” “Nesbitt,” and “Fry” using the same cDNA samples used for sequencing and 2 sets of primers. The first primer set consisted of forward primer 5′-ATGGTGGTGAAGGTGTATGGTG-3′ and reverse primer 5′-TCAAGAAGCAAGGTTCATGACTTTC-3′ and the second primer set consisted of forward primer 5′-AATGGAAGATGGTGGTGAAG-3′ and reverse primer 5′-GGATCTCAAGAAGCAAGGTT-3′. No clear bands were amplified from any of the cDNA samples using either primer set with varying PCR conditions. Synthetic plasmids for the cDNA sequences of *VrGSTF12* from “Trayshed” and “Noble” genome assemblies were ordered from (IDT Integrated DNA Technologies, Coralville, IA, USA). We then attempted to isolate *VrGSTF12* sequences from the synthetic plasmids and cDNA synthesized from berry skins of (AM-70, NC67A015_26, “Noble,” “Carlos,” “Fry,” and “Summit”) using both primer sets.

Each PCR amplification for *VrGST4* and *VrGSTF12* was performed in a total reaction volume of 25 µl containing 50 ng of DNA template, 1× GenScript PCR buffer (10 mM Tris- HCl, 50 mM KCl, 1.5 mM MgCl2, and 0.1% Triton X-100 buffer), 0.2 mM dNTPs, 0.2 µM each primer, and 0.05 U of GenScript Taq polymerase (GenScript Corporation, Piscataway, NJ, USA). The amplification reactions were carried out in a Thermal cycler (Bio-Rad Laboratories Inc., Hecules, CA, USA). Conditions for *VrGST4* were initial denaturation at 94°C for 3 min, 35 cycles of denaturation at 94°C for 1 min, annealing at 52°C or 54°C for 30 s, extension at 72°C for 1 min, and final extension at 72°C for 5 min. Isolation of *VrGSTF12* sequences was performed with initial denaturation at 94°C for 2 min, 35 cycles of denaturation at 94°C for 30 s, annealing at 55.5°C for 30 s, extension at 72°C for 30 s, and final extension at 72°C for 5 min. Amplified products were purified using the QIAquick PCR purification kit (Qiagen, Valencia, CA, USA) and sequenced at the University of Arkansas for Medical Sciences (UAMS) Sequencing Core Facility using a 3,500 Genetic Analyzer (Applied Biosystems, Foster City, CA, USA).

Sequence alignment and analysis of the PCR products and *VrGST4* and *VrGSTF12* sequences from “Trayshed,” “Noble,” and “Regale” were performed using Multalin (Corpet, 1988) software. Samples were sequenced in both the 5′ and 3′ directions to detect any errors from PCR and/or sequencing. For further confirmation of the candidate *GST4* and *GSTF12* gene isolations, sequences were compared with the “Trayshed” genome assembly and known *GST* sequences from related species using BLAST. Deduced protein sequences of VrGST4 from cDNA from “Fry,” “Black Beauty,” “Supreme,” and “Nesbitt” berry skins and VrGSTF12 from the “Trayshed” and “Noble” genome assemblies were aligned with GSTs associated with anthocyanin pathway from diverse plant species using ClustalW (Thompson *et al.* 1994). Based on the alignment, a phylogenetic tree was generated using PhyML v20160115 (https://www.genome.jp/tools/ete/; accessed 2021 Nov 10).

### RNA sequencing and gene expression analysis

Eighteen sequencing libraries were prepared using the Illumina TruSeq RNA sample preparation kit v.2 (Illumina, CA, USA) and RNA extracted from 3 biological replicates of berry skins from 3 black-fruited genotypes (AM-70, NC67A015_26, and “Noble”) and 3 bronze-fruited genotypes (“Carlos,” “Fry,” and “Summit”) collected at véraison. Size and purity of the cDNA libraries were assessed using the High Sensitivity DNA kit on a Bioanalyzer 2100 (Agilent Technologies, CA, USA). cDNA libraries were sequenced using an Illumina NextSeq sequencer (DNA Technologies Core, University of California, Davis, CA, USA) as 85-bp single-end reads.

Removal of adapter sequences, quality trimming, and length filtering was performed using Trimmomatic v.0.36 (Bolger *et al.* 2014) and the following settings: LEADING : 3 TRAILING : 3 SLIDINGWINDOW : 10:20 MINLEN : 36. Poly-G were removed with cutadapt (Martin 2011). RNA-seq reads were aligned onto the diploid “Trayshed” genome using HISAT2 v.2.1.0 (Kim *et al.* 2015) and the following settings: -end-to-end –sensitive –k 100. The parameter –k 100 allows the aligner to report up to 100 alignments per read. Default settings for scoring penalties were implemented in HISAT2 (–6 for a mismatch; –5 for a gap open; –3 for a gap extend). HISAT2 reports alignments based on the overall alignment score, which is calculated by summing together scoring penalties and matches. Among all the alignments, we observed a maximal edit distance (NM) of 8, which corresponds to a threshold of mismatch of 0.094. Alignments were visualized using integrative genomics viewer (IGV) v.2.4.14 (Robinson *et al.* 2011). Transcript abundance was quantified using Salmon v.1.5.1 (Patro *et al.* 2017) with the options: –gcBias –seqBias –validateMappings. Salmon v.1.5.1 uses an expectation-maximization approach to deal with multimapped reads. The transcriptome index file was built with “Trayshed” coding sequences (Cochetel *et al.* 2021), a k-mer size of 13, and the “Trayshed” genome as decoy. Significant differences between gene expression [transcript per Million (TPM)] of *VrGST4* among the 6 genotypes were evaluated with Kruskal–Wallis test followed by post hoc Dunn's test (*P*-value ≤ 0.05).

### KASP genotyping assay

A KASP assay for a single SNP identified in the *VrGST4* sequence was designed using 2 forward primers (5′-CTCGCTGATCTGAGTCATCTTCT-3′ and 5′-CTCGCTGATCTGAGTCATCTTCC-3′) and 1 common reverse primer (5′-CCAGCTTCCTTCACCAAGTTTCTGAT-3′). The 2 allele-specific forward primers were designed with a unique tail sequence labeled with universal FRET (fluorescence resonant energy transfer) cassettes, FAM or HEX dye. Genotyping was performed on 126 selections from the UA and UGA breeding programs, 76 diverse cultivars, 39 progeny from the Ga. 8-1-313 × “Southern Home” mapping population, 163 progeny from the “Supreme” × “Nesbitt” mapping population, and 157 progeny from the “Black Beauty” × “Nesbitt” mapping population at LGC’s genotyping service (LGC Genomics, Beverly, MA, USA). The KASP assay for each sample was conducted in a 4 μl reaction which included 2 μl low ROX KASP master mix, 0.106 μl of primer mix (0.318 μl of each primer at final concentration) and 2 μl of 10–25 ng μl^−1^ genomic DNA. The PCR conditions were an initial denaturation step at 94°C for 15 min, followed by 10 cycles of touch down PCR with annealing temperatures decreasing from 68°C to 60°C dropping at the rate of 0.8°C per cycle. This was followed by 30 cycles of denaturation at 94°C for 20 s and primer annealing at 57°C for 1 min. After the amplification reaction, PCR fluorescent endpoint data were read using the Light Cycler 480 Real-Time PCR System (Roche, Germany). The fluorescence signals measured from each sample were used to create a cluster plot in the R package ggplot2 (Wickham 2016).

### Berry color and anthocyanin profiling

Berry samples for anthocyanin profiling were harvested from the 4 muscadine cultivars (“Black Beauty,” “Fry,” “Nesbitt,” and “Supreme”) and a subset of progeny from the 2 biparental mapping populations (“Black Beauty” × “Nesbitt” and “Supreme” × “Nesbitt”). Forty-eight progeny, 16 from each genotype class (CC, CT, or TT), were randomly selected from each population for anthocyanin profiling. Skin color at the equator was measured using a CR400 colorimeter (Konica Minolta, Ramsey, NJ, USA) for 5 berries from each genotype. Color was measured as L* a* b* coordinates and were transformed into chroma (C*) and hue angle (h°) using the equations: C* = (a*2 + B*2)1/2 and h° = tan^–1^(b*/a*) (McGuire 1992). Phenolics from the 5 berry skins from each sample were extracted following Cho *et al.* (2004) and total anthocyanin content was determined using the pH differential method (Giusti and Wrolstad 2001).

A subset of 5 randomly selected samples from each genotype class in each population were evaluated for individual anthocyanins using high-performance liquid chromatography (HPLC) following methods described in Barchenger *et al.* (2015). Anthocyanin peaks were quantified at 510 nm and the data were expressed as mg cyanidin-3-glucoside equivalents per 100 g fresh fruit weight. All anthocyanin and berry color data were analyzed using ANOVA in SAS 9.4 (Cary, NC) and mean separation tests were conducted using Tukey’s Honestly Significant Difference.

## Results

### Identification and characterization of VrGST4 and VrGSTF12

The *V. vinifera* genome was initially used as the reference to identify potential candidate gene(s) for muscadine berry color variation within the 0.8 Mbp locus identified by Lewter *et al.* (2019). Twenty-one genes annotated with known or putative function were identified between 11.1 and 11.9 Mbp on chromosome 4 of the 12X.0 version of the PN40024 *V. vinifera* reference genome. Among those, 2 genes annotated as *VvGST4* and another a phi-type GST-coding gene were associated with the anthocyanin biosynthetic pathway (Alfenito *et al.* 1998; Conn *et al.* 2008). Homologs of *VvGST4* and the other phi-type GST-coding gene were subsequently identified in the “Trayshed” (Cochetel *et al.* 2021) and “Noble” (Park *et al.* 2022) *V. rotundifolia* genome assemblies and designated as *VrGST4* and *VrGSTF12*. Raw genomic sequence data from “Regale” were aligned to the “Trayshed” assembly to obtain 100% coverage of both *VrGST4* and *VrGSTF12* with 22x and 26x read depth, respectively.

**Fig. 1.**
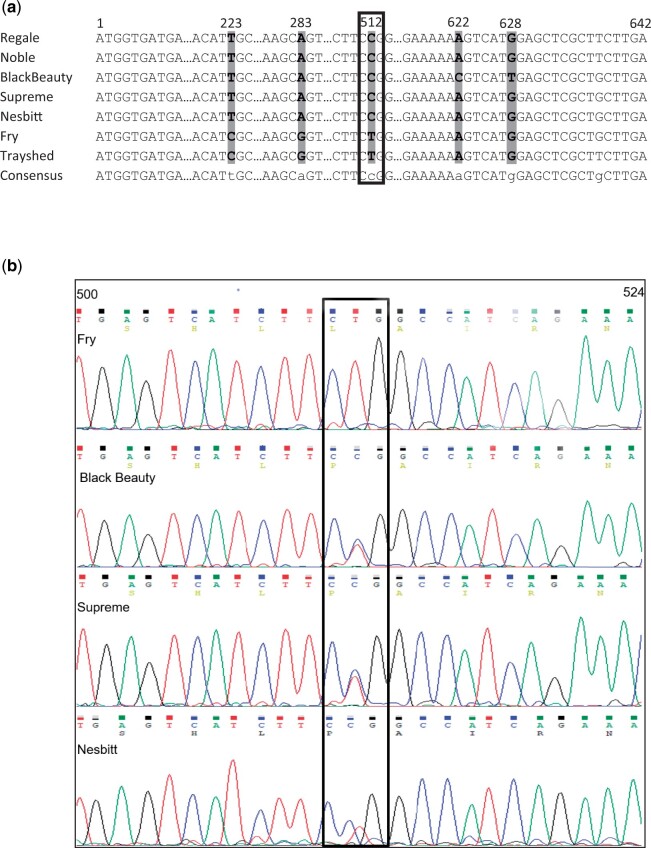
VrGST4 cDNA analysis. a) cDNA alignment of black (“Regale,” “Noble,” “Black Beauty,” “Supreme,” “Nesbitt”) and bronze (“Fry,” “Trayshed”) muscadines. Dots represent the sequences with no polymorphisms. SNPs are represented by gray highlights and the nonsynonymous SNP is represented by a black box. b) Chromatograms of the nonsynonymous SNP region in the sequences of “Black Beauty,” “Supreme,” “Nesbitt,” and “Fry” with the CTG/CCG codon highlighted in the black box. Peaks represent the corresponding nucleotide in the sequence. Numbers above the sequence indicate nucleotide positions.

A 395 bp genomic DNA fragment was amplified from the leaf tissues of black and bronze-fruited muscadines using the *VvGST4* sequence information (Supplementary Fig. 1). The amplified 395 bp sequence from muscadine showed an identity of 98% with *VvGST4* and *V. amurensis* (*VaGST4*; GenBank Accession No. FJ645770.1) sequences suggesting that *VrGST4*, is a likely candidate gene for berry color variation in muscadine (Supplementary Table 1). A full-length *VrGST4* cDNA of 642 bp was then amplified from berry skins of a bronze-fruited muscadine cultivar, “Fry,” and 3 black-fruited muscadine cultivars, “Supreme,” “Black Beauty,” and “Nesbitt.” These cDNA sequences were compared to *VrGST4* sequences from “Trayshed,” “Noble,” and “Regale.” “Trayshed” was expected to be homozygous for the bronze allele because it is a staminate genotype that produces 100% bronze-fruited progeny when used as a pollen parent in crosses with bronze-fruited pistillate cultivars (Andy Walker, Personal Communication). In contrast, “Noble” and “Regale” are black-fruited cultivars. Five SNPs were detected among the 7 *VrGST4* sequences, with only 1 nonsynonymous polymorphism (C/T) identified toward the 3′ end of the gene at position 512 (Fig. 1a). Figure 1b shows the chromatograms of the nonsynonymous SNP region in the bronze and black muscadine sequences with CCG codon identified in the black muscadines and CTG codon in the bronze muscadine. The overlapping signals for C or T at position 512 in “Supreme,” “Black Beauty,” and “Nesbitt” indicates the presence of both CCG and CTG alleles in these cultivars.

**Fig. 2.**
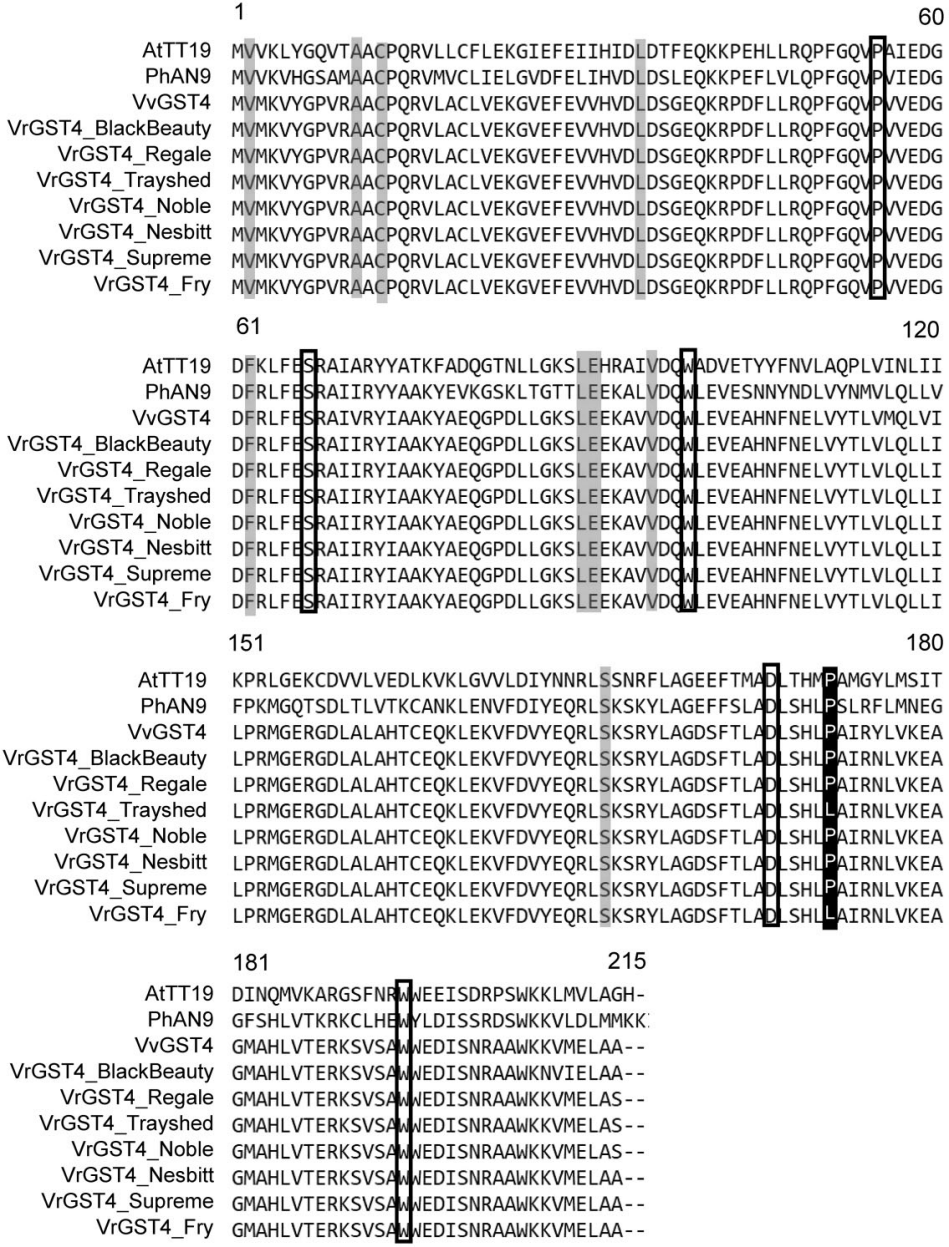
VrGST4 protein alignment. Comparison of VrGST4 from postvéraison berry skins of “Fry,” “Supreme,” “Black Beauty,” and “Nesbitt” muscadine cultivars with predicted protein sequences of “Trayshed,” “Noble,” and “Regale” from genome assemblies and resequencing data, and anthocyanin-related GST sequences from Arabidopsis (AtTT19), Petunia (PhAN9), and *V. vinifera* (VvGST4). Numbers above the sequence alignment represent amino acid positions. Black shaded region indicates the proline to leucine mutation at position 171. Gray shaded regions indicate amino acid residues specific to anthocyanin-related GSTs (Kitamura *et al.* 2012). Black boxes indicate amino acid residues suggested previously to have high homology in anthocyanin-related GSTs (Alfenito *et al.* 1998).

The deduced protein from the full-length *VrGST4* sequence of both bronze and black-fruited muscadines had 213 amino acids with a predicted molecular weight of 24.2 kDa in all 7 muscadine cultivars. A BLASTp search of the VrGST4 protein sequences was performed to compare the sequence similarity with known GST4 proteins from other species (Fig. 2). VrGST4 protein had 98%, 65%, and 56% sequence identity with VvGST4 (NP_001267869) from *V. vinifera*, PhAN9 (CAA68993) from *P.hybrida*, and AtTT19 (AED92398) from *A.thaliana*, respectively. Protein alignment indicated that the C/T SNP identified in *VrGST4* cDNA corresponded to a proline to leucine mutation, thereby confirming the presence of an intragenic SNP marker within the candidate *VrGST4* gene. Leucine was present at position 171 in both bronze muscadines (“Trayshed” and “Fry”), whereas proline was present at this position in VvGST4, PhAN9, AtTT19, and VrGST4 proteins of black muscadines (“Black Beauty,” “Supreme,” “Nesbitt,” “Regale,” and “Noble”).

Initial attempts to amplify the full-length *VrGSTF12* cDNA sequence from berry skins of “Fry,” “Supreme,” “Black Beauty,” and “Nesbitt” were unsuccessful despite attempts with multiple primer sets and a wide array of PCR conditions. The publication of the “Trayshed” (Cochetel *et al.* 2021) and “Noble” (Park *et al.* 2022) genome assemblies enabled comparison of cDNA sequences from bronze and black muscadine genotypes. Four SNPs were detected among the *VrGSTF12* sequences for “Trayshed,” “Noble,” and “Regale,” with 2 nonsynonymous polymorphisms identified at positions 311 (A/C) and 589 (A/G) (Supplementary Fig. 2). “Trayshed” was homozygote for the A allele, “Noble” was homozygote for the C allele, and “Regale” was heterozygous for the nonsynonymous SNP at position 311. Both “Trayshed” and “Regale” were homozygous for the G allele while “Noble” was heterozygous at position 589.

The deduced protein from the full-length VrGSTF12 sequences of “Trayshed,” “Noble,” and “Regale” all had 213 amino acids (Supplementary Fig. 3). Protein alignment indicated that the A/C SNP identified in VrGSTF12 cDNA corresponded to a glutamic acid to alanine to mutation at position 104 and the A/G SNP corresponded to a glutamic acid to lysine mutation at position 197. Glutamic acid was present at position 104 for *V. vinifera* (PN40024) and “Trayshed,” a bronze-fruited muscadine, whereas alanine was present in 1 allele of “Regale” and both alleles of “Noble.” At position 197, glutamic acid was present in *V. vinifera* (PN40024), “Trayshed,” and “Regale,” while “Noble” was heterozygous with alleles with glutamic acid and lysine.

**Fig. 3.**
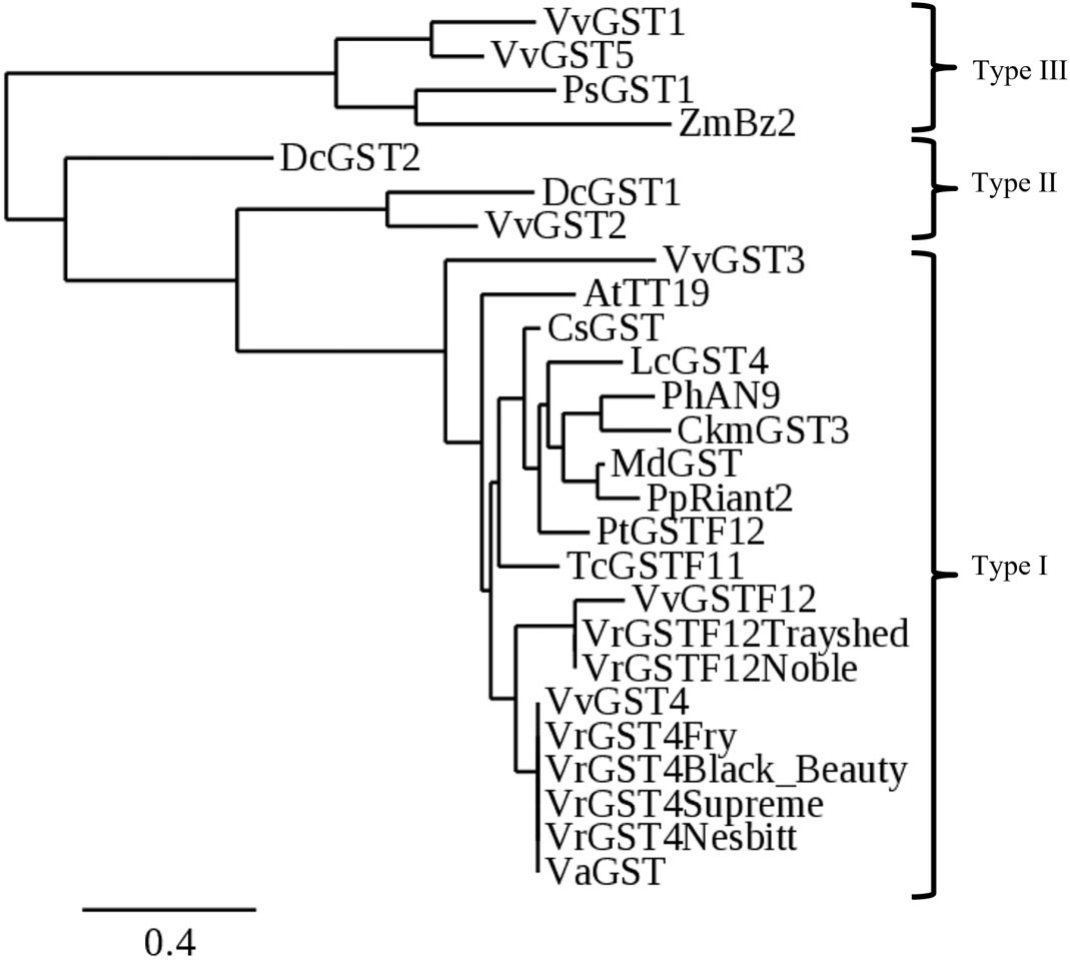
Phylogenetic tree derived from deduced protein sequences of *VrGST4* from bronze (“Fry”) and black (“Black Beauty,” “Supreme,” and “Nesbitt”) muscadines, and *VrGSTF12* from bronze (“Trayshed”) and black (“Noble”) muscadine genomes relative to protein sequences of known GSTs from other species retrieved from GenBank. GST classification is represented by Type I, II, and III classes. At, *A. thaliana* (AtTT19, AED92398); Ckm, *Cyclamen* sp. (BAM14584); Cs, *C. sinensis* (ABA42223); Dc, *Dianthus caryophyllus* (DcGST2, AAA51450; DcGST1, AAA72320); Lc, *L. chinensis* (KT946768); Md, *Malus domestica* (AEN84869); Ph, *P. hybrida* (CAA68993); Pp, *Prunus persica* (KT312848); Ps, *Papaver somniferum* (AAF22517); Pt, *Populus trichocarpa* (XP006372485); Tc, *Theobroma cacao* (EOY03123); Va, *Vitis amurensis* (ACN38271); Vr, *Vitis rotundifolia* (VrGST4Fry, MT678460; VrGST4Supreme, MT678462; VrGST4BlackBeauty, MT678461; VrGST4Nesbitt, MT678463; VrGSTF12Trayshed, VITMroTrayshed_v1.1_EVM.ver0.0.g1.04.943.1.t01; VrGSTF12Noble); Vv, *V. vinifera* (VvGST1, AAN85826; VvGST2, ABK81651; VvGST3, ABO64930; VvGST4, NP001267869; VvGST5, ABL84692; VvGSTF12, RVW61068); Zm, *Z. mays* (AAA50245).

A phylogenetic tree derived from the deduced VrGST4 and VrGSTF12 protein sequences of bronze and black muscadines, the “Trayshed” muscadine genome assembly and other known GSTs indicated that VrGST4 and VrGSTF12 belonged to the Phi group of GST proteins (Fig. 3). Results showed that VrGST4 from both bronze and black muscadines is homologous to VvGST4 (Conn *et al.* 2008; Pérez-Díaz *et al*. 2016) from *V. vinifera* and VaGST4 from *V. amurensis*, and VrGSTF12 from both bronze and black muscadines is homologous to VvGSTF12 from *V. vinifera*. VrGST4 and VrGSTF12 alleles grouped in the same clade as PhAN9 (Alfenito *et al.* 1998; Mueller *et al.* 2000), AtTT19 (Kitamura *et al.* 2004), MdGST (Jiang *et al.* 2019) and other Phi group GSTs such as CkmGST3 of *Cyclamen* sp. (Kitamura *et al.* 2012), CsGST of *Citrus sinensis* (Licciardello *et al.* 2014), and LcGST4 of *Litchi chinensis* (Hu *et al.* 2016). Other anthocyanin-related GSTs belonging to the Tau group, ZmBz2 of *Z.mays* (Marrs *et al.* 1995) and VvGST1 of *V. vinifera* (Pérez-Díaz *et al*. 2016), clustered separately.

### Expression of *VrGST4*, *VrGSTF12*, and *VrMybA1* in berry skins

**Fig. 4.**
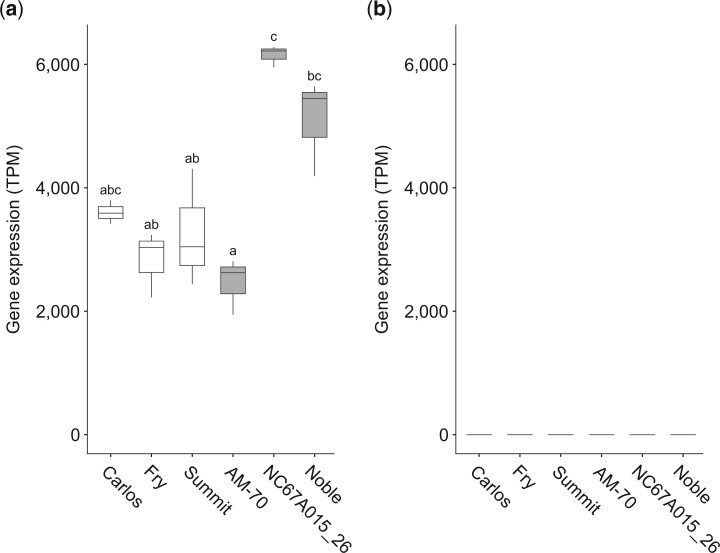
Gene expression of *VrGST4* (a) and *VrGSTF12* (b) in berries of 3 bronze (“Carlos,” “Fry,” and “Summit”) and 3 black (AM-70, NC67A015_26, and “Noble”) muscadine genotypes. Gene expression is represented as Transcript per Million (TPM). *n* = 3. Significant gene expression differences are indicated by different letters (Kruskal–Wallis test followed by post hoc Dunn's test; *P*-value ≤ 0.05).

On average, 20.6 ± 3.3 million RNA sequencing (RNA-seq) reads were generated for each sample (Supplementary Table 2). Alignment of the RNA-seq data on the “Trayshed” genome confirmed the presence of a heterozygous SNP (C/T) at position 512 of the coding sequence of *VrGST4* in all the black muscadines, AM-70, NC67A015_26, and “Noble” (Supplementary Fig. 4). The bronze genotypes, “Carlos,” “Fry,” and “Summit,” were all confirmed to be homozygous for the T allele at position 512. Gene expression analysis showed that *VrGST4* was highly expressed in all genotypes (Fig. 4a), whereas the gene expression level of *VrGSTF12* was extremely low (<0.5 TPM; Fig. 4b). Alignments of the RNA-seq data at the *VrGSTF12* gene locus showed that short-sequencing reads aligned only on 1 exon and not on the entire gene body (Supplementary Fig. 5). In addition, gene splicing structure was not supported by any read. This suggests that the observed alignments are not specific to the *VrGSTF12* gene, and that *VrGSTF12* is not expressed in muscadine berry skins of black or bronze genotypes at véraison. The lack of expression of *VrGSTF12* in muscadine berry skins is also supported by sequencing of PCR products. We failed to obtain PCR products or sequence data from cDNA synthesized from berry skins at véraison from “Black Beauty,” “Supreme,” “Nesbitt,” AM-70, NC67A015_26, “Noble,” “Carlos,” “Fry,” or “Summit” muscadines with multiple primer combinations and PCR conditions. However, we were able to use the same primers and PCR conditions with synthetic plasmids for the predicted coding sequences of *VrGSTF12* from “Trayshed” and “Noble” assemblies to isolate sequences that aligned to the CDS sequence of *VrGSTF12* (VITMroTrayshed_v1.1_EVM.ver0.0.g1.04.943.1.t01) with 99% or greater identity (Supplementary Table 3).

Gene expression analysis also confirmed that *VrMybA1* (VITMroTrayshed_v2.0.hap1.chr02.ver2.0.g024710) was highly expressed and well-conserved in all 6 muscadine grape genotypes (Supplementary Fig. 6). Alignment of the RNA-seq data on the “Trayshed” genome indicated the presence of 3 nonsynonymous SNPs within the coding sequence of *VrMybA1* among the 6 genotypes. “Trayshed,” NC67A015_26, “Carlos,” and “Summit” all had identical coding sequences. AM-70 was heterozygous for all 3 nonsynonymous SNPs at positions 112 (G/A), 512 (T/C), and 557 (C/T). “Fry” was also heterozygous for the C/T SNP at position 557 and “Noble” was homozygous for the C allele at position 512. The nonsynonymous SNPs corresponded to alanine to tyrosine, leucine to proline, and alanine to valine substitutions at amino acid positions 38, 170, and 186, respectively.

### Validation of the *VrGST4* KASP marker in diverse genotypes

**Fig. 5.**
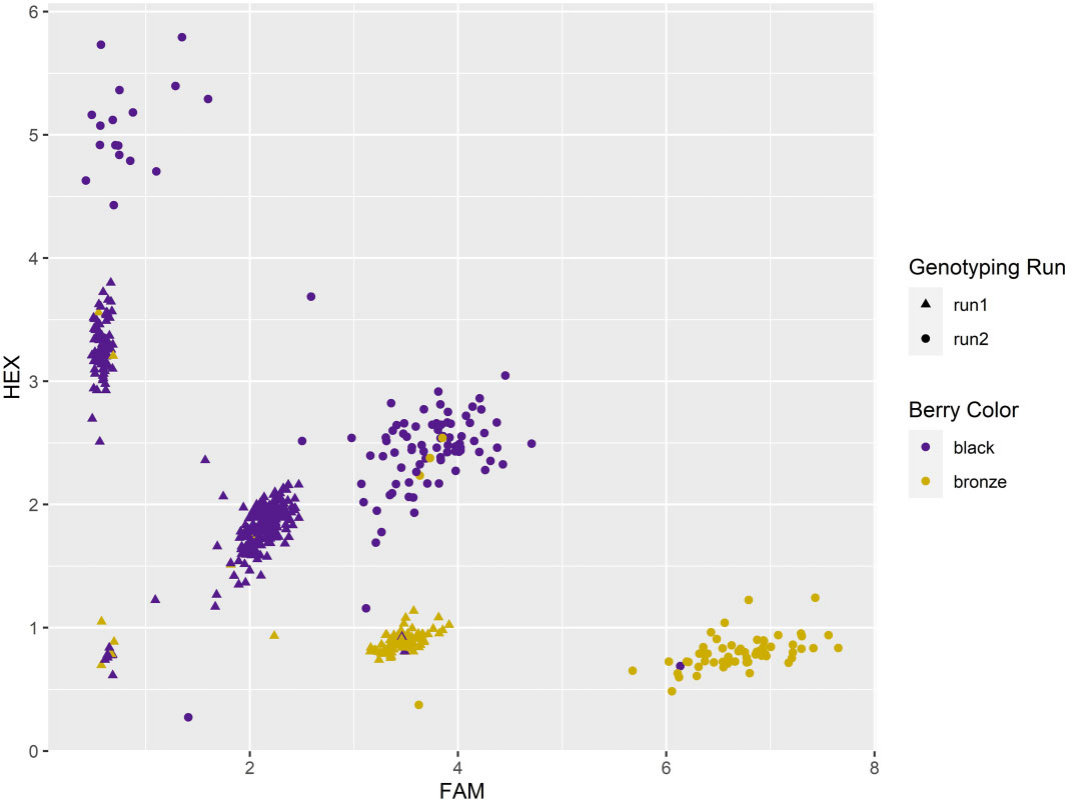
KASP genotyping assay cluster plot for an intragenic SNP (C/T) marker of *VrGST4* used to evaluate 546 muscadine genotypes. Each data point on the cluster plot represents the fluorescence signal from an individual sample and is color coded according to the berry color associated with genotype. The data points generated during the first and second sequencing runs are coded as triangles and circles, respectively. FAM fluorescence values associated with the T allele were plotted on the *x*-axis and HEX fluorescence values associated with the C allele were plotted on the *y*-axis for each sample.

In order to further validate the association of the intragenic C/T SNP in *VrGST4* with berry color in diverse muscadine genotypes, a KASP genotyping assay was performed on 359 progeny from 3 mapping populations, 126 selections from the UGA and UGA breeding programs, and 76 cultivars (Supplementary Table 4). The results are depicted as a cluster plot (Fig. 5) showing 3 clusters of data points representing homozygous black (CC), heterozygous black (CT), and bronze-fruited (TT) genotypes. Association of KASP marker data with berry color from all the genotypes revealed that CC and CT genotypes associated with the black phenotype whereas the TT genotype associated with the bronze phenotype. Cultivars and breeding selections classified as “red-fruited” grouped with the black (CT and CC) genotypes, while those with slightly pink berry color grouped with the bronze (TT) genotypes. The marker reaction failed for 17 of 561 samples and no genotype was predicted. The KASP marker correctly predicted berry color in 534 of the 544 successful reactions. New leaf tissue was collected from 6 of the vines with phenotype data that did not match the KASP genotype prediction and the full-length *VrGST4* fragment was amplified from those samples. In 5 of 6 cases, the new sequence data matched the berry color phenotype (Supplementary Table 4).

### Anthocyanins and berry color

**Fig. 6.**
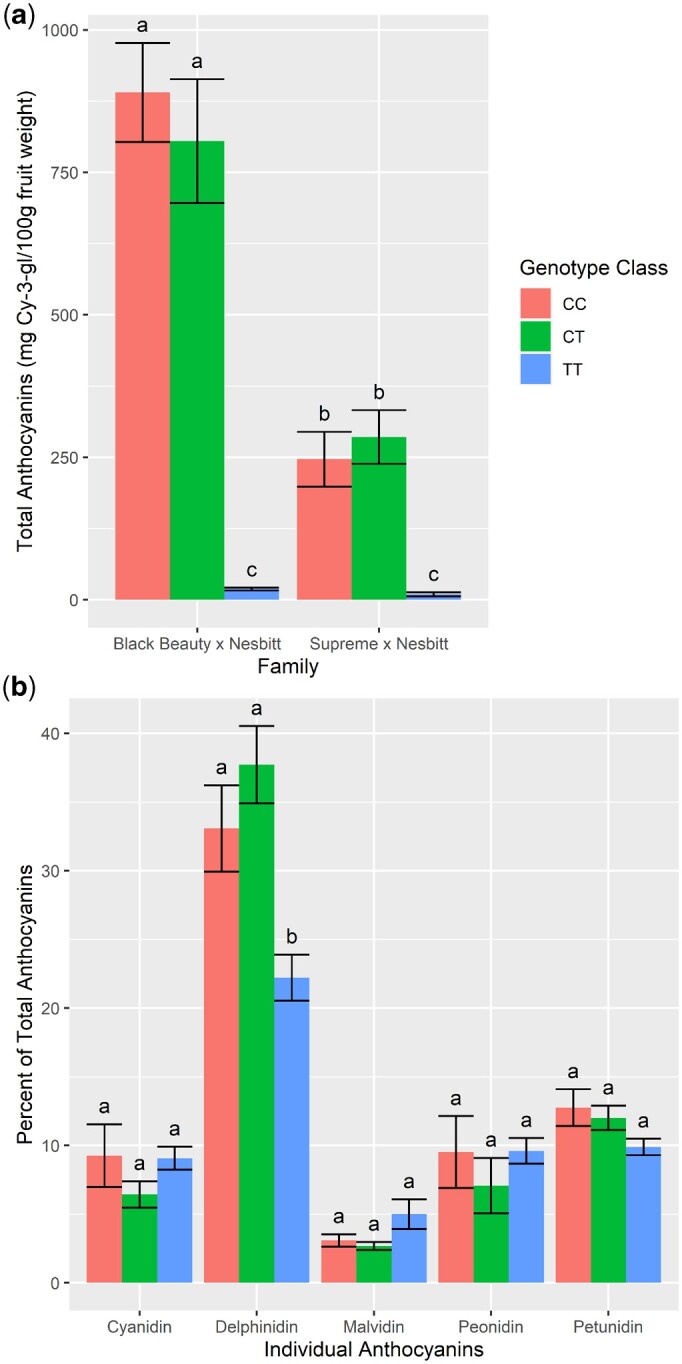
Anthocyanin profiling of postvéraison berry skins from black (CC & CT) and bronze (TT) muscadine genotypes from the “Supreme” × “Nesbitt” and “Black Beauty” × “Nesbitt” mapping populations. a) Total anthocyanin content from each population and b) Individual anthocyanins represented as an average of both populations. Error bars represent standard error of means.

Total anthocyanin content was estimated in a subset of progeny from the “Supreme” × “Nesbitt” and “Black Beauty” × “Nesbitt” mapping populations to determine gene action and allele dosage effects (Fig. 6a). Anthocyanin content was higher in homozygote and heterozygote black genotypes of both populations compared to bronze genotypes. Average anthocyanin content across black-fruited (CC and CT) genotypes was higher in the “Black Beauty” × “Nesbitt” population (847.55 mg.100 g^−1^ fresh wt) compared to the “Supreme” × “Nesbitt” population (265.98 mg.100 g^−1^ fresh wt). No significant differences in total anthocyanin content were discovered between the homozygote (CC) and heterozygote (CT) black genotypes from either mapping population. While in the “Supreme” × “Nesbitt” population, CC and CT genotypes had an average of 263.76 and 265.4 mg.100^−1^ g fresh weight, respectively, the CC and CT genotypes of “Black Beauty” × “Nesbitt” population averaged 890.19 and 883.1 mg.100 g^−1^ fresh weight, respectively. The TT genotypes averaged 9.43 and 18.64 mg.100 g^−1^ fresh weight in “Supreme” × “Nesbitt” and “Black Beauty” × “Nesbitt” populations, respectively. These results imply dominant gene action for *VrGST4*.

The composition of individual anthocyanins from a smaller subset of progeny in each mapping population was estimated using HPLC. Results are presented as percent of the total anthocyanins averaged across both mapping populations for each individual anthocyanin (Fig. 6b). The percent of 5 individual anthocyanins, cyanidin, delphinidin, petunidin, peonidin, and malvidin, was determined from the 3 genotype classes (CC, CT, and TT) in both mapping populations. Delphinidin was the most abundant anthocyanin, making up 22.2%–37.7% of total anthocyanins in all 3 genotype classes of both populations. This was followed by petunidin (9.9–12.7%), peonidin (7.1–9.6%), cyanidin (6.4–9.3%), and malvidin (2.7–5.0%). Petunidin, peonidin, cyanidin, and malvidin composition were similar among the 3 genotype classes. The bronze-fruited (TT) genotypes had slightly lower percent delphinidin composition compared to black-fruited genotypes (CC and CT).

**Fig. 7.**
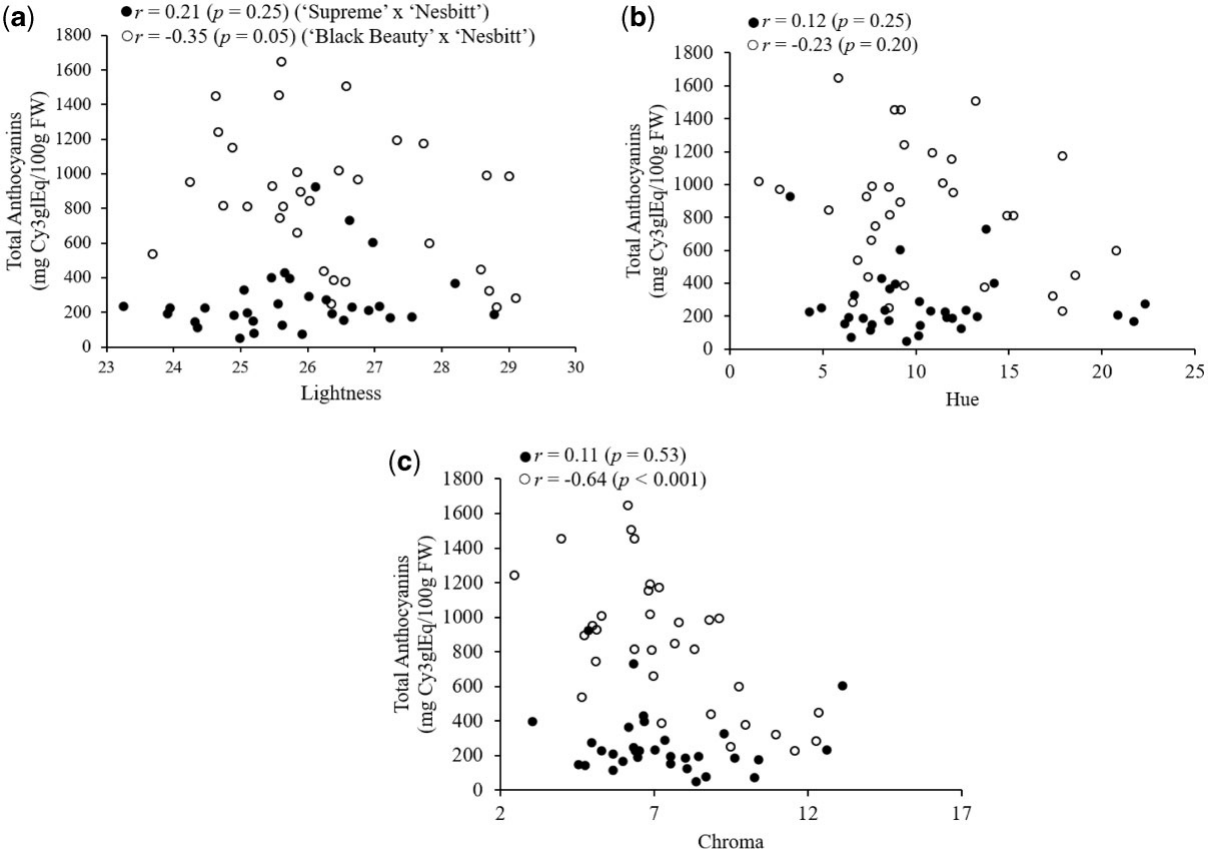
Correlation (*r*) of muscadine berry color with total anthocyanin content in postvéraison berries of black-fruited (C:C and C:T) genotypes from the “Supreme” × “Nesbitt” and “Black Beauty” × “Nesbitt” mapping populations. Color coordinates; a) Lightness, b) Hue, and c) Chroma.

There was no significant difference in any of the berry color attributes (lightness value, hue angle, and chroma) between CC and CT genotypes in either mapping population (Data not shown). Berry color of the black-fruited progeny (CC or CT genotypes) from the mapping populations was measured to determine the possible associations between berry color attributes (lightness value, hue angle, and chroma) with total anthocyanins (Fig. 7). The average lightness (25.81), chroma (7.27), and hue angle (10.30°) in black-fruited progeny from the “Supreme” × “Nesbitt” population were comparable to those of 26.39, 7.41, and 10.44°, respectively, in the “Black Beauty” × “Nesbitt” population. There was no significant correlation between total anthocyanins and lightness value (*P=* 0.25), hue angle (*P=* 0.35), or chroma (*P=* 0.53) in the “Supreme” × “Nesbitt” population. In the “Black Beauty” × “Nesbitt” population, there was no significant correlation between total anthocyanins and lightness (*P=* 0.05) or hue angle (*P=* 0.20). However, total anthocyanins were negatively correlated with chroma in the “Black Beauty” × “Nesbitt” population (*r* = −0.64, *P<* 0.001).

## Discussion

Lewter *et al.* (2019) developed the first saturated genotyping-by-sequencing-based linkage maps of muscadine grapes using 2 biparental mapping populations with “Black Beauty” or “Supreme” as the female parent and “Nesbitt” as the common male parent. These dense linkage maps were used to map the muscadine berry color locus to a 0.8 Mbp region on chromosome 4 of *V. vinifera*. Variation in berry color and anthocyanin content in *V. vinifera* is controlled by a well-documented MYB gene cluster located on chromosome 2 (Kobayashi *et al.* 2004; This *et al.* 2007; Fournier-Level *et al.* 2009; Myles *et al.* 2011). Thus, the findings of Lewter *et al.* (2019) suggested that a different gene(s) within the flavonoid biosynthetic pathway was responsible for variation in berry color in muscadine grapes and that a candidate gene associated with anthocyanin accumulation and sequestration could be identified within the 11.1–11.9 Mbp interval on chromosome 4. In the present study, we identified *VrGST4* and *VrGSTF12* as possible candidates for berry color in muscadine grapes located within the 0.8 Mbp berry color locus.

GSTs belong to a wide ubiquitous family of glutathione transferase enzymes found in bacteria, fungi, animals, and plants (Edwards *et al.* 2000). In plants, GSTs are known to perform diverse functions in the cells including defense mechanisms against pathogens, herbicide detoxification, and in pathways related to biosynthesis and detoxification of secondary metabolites such as anthocyanins (Dixon *et al.* 1998; Monticolo *et al.* 2017). GSTs can be broadly categorized into those with catalytic activity and/or ligandin activity. Catalytic activity of GSTs is used for conjugation of xenobiotic substrates with the tripeptide glutathione (GSH) and function in the detoxification of herbicides. GSTs with ligandin activity function in a noncatalytic role by acting as carrier proteins for shuttling of several endogenous compounds including vacuolar sequestration of anthocyanins (Dixon *et al.* 2002; Monticolo *et al.* 2017). GSTs from many species have been demonstrated to have a ligandin activity for the transport of anthocyanins from cytosol to vacuoles. In *V. vinifera*, 5 GST genes (*VvGST1* to *VvGST5*) have been identified that are associated with the flavonoid biosynthetic pathway (Conn *et al.* 2008; Pérez-Díaz *et al*. 2016). While *VvGST1* and *VvGST4* were associated with anthocyanin transport in berries, *VvGST3* was found to play a major role in proanthocyanidin transport in seeds.

GSTs are classified into 6 classes, Tau, Phi, Zeta, Theta, Lambda, and Dhar, based on the identity of amino acid sequence. Tau and Phi are plant-specific classes due to their greater representation in terms of number of sequences (Edwards and Dixon 2005). Many GSTs have been identified in plants such as *Arabidopsis* (53 GSTs; Sappl *et al.* 2009), rice (59 GSTs; Soranzo *et al.* 2004), *C.sinensis* (61 GSTs; Licciardello *et al.* 2014), and *L.chinensis* (139 GSTs; Hu *et al.* 2016). Phylogenetic analysis based on deduced protein sequences (Fig. 3) revealed that *VrGST4* of both bronze and black muscadine cultivars clustered closely with GSTs from *Vitis* (*VaGST* and *VvGST4*) and formed a subclade with *VrGSTF12* and *VvGSTF12*. All GST4 and GSTF12 alleles from *Vitis* sp. were closely related to Phi group GSTs from other dicotyledonous plants in the genera *Arabidopsis*, *Petunia*, *Citrus*, *Malus*, and *Litchi*. The Phi group GSTs were previously reported to function as anthocyanin transporters in the flavonoid biosynthetic pathway (Conn *et al.* 2008; Pérez-Díaz *et al*. 2016; Alfenito *et al.* 1998; Mueller *et al.* 2000; Kitamura *et al.* 2004; Hu *et al.* 2016; Jiang *et al.* 2019). Both GST4 and GSTF12 genes are implicated in anthocyanin transport and vacuolar anthocyanin sequestration. The Arabidopsis *GSTF12* gene is also known as *TT19* due to the Transparent Testa 19 phenotype resulting from a loss-of-function mutation in *AtGSTF12* (Dixon and Edwards 2010).

GSTs have been classified as Type I, II, or III based on their intron: exon structure (Droog 1997). According to this classification, *ZmBZ2*, *VvGST1*, and *VvGST5*, which are Tau class GSTs grouped as Type III class with 2 exons and 1 intron (Marrs 1996; Conn *et al.* 2008). *DcGST1* and *DcGST2* from carnation grouped as Type II GSTs known to have 10 exons and 9 introns (Marrs 1996; Sasaki *et al.* 2012). *VvGST4, PhAN9*, *AtTT19* clustered in the Type I class with 3 exons and 2 introns (Marrs 1996; Conn *et al.* 2008). *VrGST4* and *VrGSTF12* both belong to the Type I class based on the predicted intron: exon structure in the “Trayshed” annotation (Cochetel *et al.* 2021). Although the Tau GSTs have a gene structure that is different from Phi GSTs, they were found to complement each other functionally in terms of anthocyanin transport. For example, *ZmBZ2* and *PhAN9* reciprocally complemented an9 and bz2 tissues in particle gun bombardment assays (Alfenito *et al.* 1998) even though they have only 12% amino acid identity. This functional complementation between monocot and dicot GST proteins suggests that they share a common ancestral gene before evolution into species-specific GSTs that transport different anthocyanin pigments (Alfenito *et al.* 1998).

Sequence analysis of *VrGST4* cDNA from the black and bronze muscadines isolated in our study and alignment of the RNA-seq data on the “Trayshed” genome revealed a single intragenic SNP (C/T) corresponding to a proline to leucine mutation in bronze muscadines (Figs. 1a and 2, Supplementary Fig. 4). Comparison of *VrGSTF12* sequences from “Trayshed,” “Noble,” and “Regale” (Supplementary Figs. 2 and 3) indicated that 2 nonsynonymous SNPs were present corresponding to a glutamic acid to alanine to mutation at position 104 and the A/G SNP corresponded to a glutamic acid to lysine mutation at position 197. The allele at position 197 was unlikely to cause bronze berry color because *V. vinifera* (PN40024), bronze muscadine “Trayshed,” and the black muscadine “Regale” were all homozygous for glutamic acid, while only “Noble” had a lysine allele. However, both black-fruited muscadines, “Noble” and “Regale” had at least 1 alanine allele at position 104, while “Trayshed” and *V. vinifera* (PN40024) were homozygous for glutamic acid. Therefore, *VrGSTF12* could not be excluded as a potential berry color candidate based on sequence comparisons alone.

RNA-Seq analysis showed that *VrGST4* was highly expressed in berry skins at véraison from both bronze- and black-fruited muscadine genotypes (Fig. 4a). In contrast the gene expression level of *VrGSTF12* was extremely low (<0.5 TPM; Fig. 4b) in all 3 biological replicates of the 6 muscadine genotypes. We also attempted to isolate the coding sequence of *VrGSTF12* from cDNA synthesized from berry skins collected from 6 black-fruited and 3 bronze-fruited muscadine genotypes and from synthetic plasmids of predicted coding sequences of *VrGSTF12* from “Trayshed” and “Noble” assemblies. We were only able to isolate sequences corresponding to *VrGSTF12* from the synthetic plasmids (Supplementary Table 3), which added further support to the idea that *VrGSTF12* is not expressed in berry skins. Taken together, the results of sequence alignments and expression analysis indicated that *VrGST4* was the more likely candidate gene for berry color in muscadine grapes.

The proline to leucine mutation in VrGST4 is located in the C-terminal alpha-helical domain, which is a hydrophobic cosubstrate binding site known as the H- site. It is positioned adjacent to the conserved GSH binding domain, known as G-site. While the G-site is very specific, accepting only GSH or other closely related gamma-glutamyl linked peptides, the H-site is known to accommodate a diverse range of substrates and ligands. Involvement of GST4 or GSTF12 in catalytic reactions at the G-site is highly unlikely due to the absence of the GSH-activating residues (serine or cysteine) (Dixon and Edwards 2010). Instead, GST4 and GSTF12 are demonstrated to be carrier proteins binding to hydrophobic xenobiotic molecules in a noncatalytic role at the H-site. This nonenzymatic ligandin activity has been documented in many plants for the binding and transport of flavonoids such as anthocyanins and proanthocyanidins (Marrs *et al.* 1995; Alfenito *et al.* 1998; Kitamura *et al.* 2004; Conn *et al.* 2008; Perez-Diaz *et al.* 2016) and several endogenous compounds including oxylipins and phytohormones such as auxin and cytokinin (Smith *et al.* 2003; Gong *et al.* 2005; Moons 2005). In bronze muscadines, the proline to leucine mutation in VrGST4 implies that it does not function in anthocyanin transport. However, VrGST4 is highly expressed in berry skins of both black and bronze muscadines at véraison, which indicates the possibility that this protein may still have other functional roles, such as the transport of alternative ligands, cell signaling and/or plant growth and development. Further research is needed through functional complementation assays to determine the role of mutated *VrGST4* in bronze cultivars.

Results from a KASP genotyping assay developed from the intragenic SNP (C/T) in *VrGST4* (Fig. 5, Supplementary Table 4) showed that the marker was able to distinguish between bronze (TT), heterozygote black (CT), and homozygote black (CC) genotypes and accurately predict berry color phenotype in a panel of 126 breeding selections, 76 cultivars, and 359 progeny from 3 mapping populations. The KASP marker prediction was inconsistent with berry color in only 10 of the 544 successful reactions. In 5 of 6 cases with inconsistencies between marker predictions and berry color, the full length *VrGST4* sequence data matched the berry color phenotype, indicating that the discrepancies between marker predictions and phenotypes were caused by errors in leaf collection among tightly spaced plants in the research vineyard (Supplementary Table 4). Leaf tissue was obtained from the USDA National Clonal Germplasm Repository for the 3 cultivars (“Chief,” “San Alba,” and “Stuckey”) with conflicting phenotype and KASP marker data. The berry color of these genotypes was obtained from historical records and not confirmed at the repository. Previous studies investigating genetic diversity and pedigrees of muscadine cultivars and germplasm accessions using simple sequence repeats (SSRs) have identified several inconsistencies between marker fingerprints and pedigree records for older cultivars held in the USDA National Clonal Germplasm Repository (Cao *et al.* 2020; Riaz *et al.* 2008). Cao *et al.* (2020) specifically reported that “Stuckey” obtained from the USDA National Clonal Germplasm Repository had SSR fingerprint data conflicting with its reported pedigree. Therefore, it is possible that the inconsistencies between KASP predictions and reported berry color phenotypes are due to identification errors in the repository. Overall, the intragenic *VrGST4* KASP marker had excellent predictive ability for berry color in a diverse set of muscadine germplasm which included many diverse historical and foundational cultivars propagated from the wild during the 19th century such as “Flowers,” “James,” “Memory,” “Thomas,” and “Scuppernong.”

Total and individual anthocyanins were measured in a subset of progeny from each mapping population to investigate whether allele dosage (additive genetic variation) at *VrGST4* plays a significant role in determining anthocyanin content in muscadine skins. There was no difference in the total anthocyanin content or berry color attributes (L, hue angle, chroma) between the homozygote and heterozygote black genotypes (Fig. 6a) in either mapping population, indicating completely dominant gene action for *VrGST4* in muscadine. In contrast, allele dosage in the MYB gene cluster plays a major role in determining anthocyanin content in *V. vinifera* and most phenotypic variation in grape anthocyanin content has been attributed to additive effects, with dominance playing a minor role (Fournier-Level *et al.* 2009). Our findings suggest that while the intragenic *VrGST4* KASP marker can be used to predict bronze or black berry color in breeding populations and distinguish homozygote and heterozygote black genotypes from one another, it is not useful for selecting progeny with high anthocyanin production for processing and nutraceutical industries.

Anthocyanin content in the skins of black-fruited muscadines has previously been shown to range from less than 100 mg.100 g^−1^ to over 500 mg.100 g^−1^ (Conner and MacLean 2013). In this study, the average anthocyanin content across black-fruited (CC and CT) genotypes was over 3 times higher in the “Black Beauty” × “Nesbitt” population (847.55 mg.100 g^−1^ fresh wt) than the “Supreme” × “Nesbitt” population (265.98 mg.100 g^−1^ fresh wt). This discrepancy in anthocyanin content of black-fruited progeny between the 2 populations could be attributed to many factors, including possible differences in ripeness on the date of harvest. The large difference between the means of the black-fruited genotypes in the 2 mapping populations also suggests that other loci in addition to *VrGST4*, including the MYB gene cluster on chromosome 2, may contribute to quantitative variation in total anthocyanin content in red and black-fruited muscadines. In this study, we found that *VrMybA1* was highly expressed in all 6 genotypes evaluated in the RNA-Seq experiment and generally well-conserved. Three nonsynonymous SNPs were discovered among these genotypes corresponding to amino acid substitutions at positions 38 (alanine to tyrosine,) 170 (leucine to proline), and 186 (alanine to valine). None of these polymorphisms were consistently associated with bronze or black phenotypes in muscadine grapes and the MYB gene cluster maps to a different chromosome than the muscadine color locus. However, it is possible that one or more of these polymorphisms may impact total anthocyanin content in black-fruited muscadines. Further investigations are needed to determine whether allelic variation in *VrMybA1* or other loci contribute to the large range in total anthocyanin content among black-fruited muscadines.

Estimation of anthocyanin content based on berry color is challenging, as color is not always a good predictor of nutraceutical content. Food color is a critical parameter used as a quality index and is most often described by measurements of lightness (brightness), chroma (degree of color saturation), and hue angle (color wheel with red, yellow, green, and blue at 0°, 90°, 180°, and 270°, respectively). We measured surface color characteristics in a representative sample from homozygote and heterozygote black genotypes of the 2 mapping populations to determine if berry color was correlated with total anthocyanin content in black muscadines (Fig. 7). Although the “Black Beauty” × “Nesbitt” population had higher anthocyanin content compared to the “Supreme” × “Nesbitt” population, average berry color parameters were similar in both populations. The negative correlation between chroma and total anthocyanins observed in the “Black Beauty” × “Nesbitt” population (Fig. 7c) may be attributed to genotype-specific differences in the epicuticular wax deposition during berry development. Spinardi *et al.* (2019) observed a similar decrease in both chroma and L* values during blueberry development as the berry skin pigmentation and cuticular wax load increased resulting in darker and less vivid color of the berry peels. Overall, our results suggest that surface color characteristics are not a good predictor of anthocyanin content in black-fruited muscadine grapes. A similar result was reported by Muthusamy *et al.* (2014), who found that color was poor indicator of beta carotene content in maize, with no significant correlation observed between color and nutraceutical content. In contrast, Palonen and Weber (2019) observed significant correlations between anthocyanin concentration and hue values and L*a*b ratios in raspberry.

Besides anthocyanin quantity, composition of individual anthocyanins also varies widely in fruits. Six anthocyanins have been detected in *V. vinifera* grapes and muscadines, among which delphinidin and cyanidin are most commonly found in muscadines (Conner and MacLean 2013). Our results from anthocyanin composition analysis in black and bronze muscadines show that delphinidin was the predominant type of individual anthocyanin in both black and bronze berries of the 2 mapping populations, which is consistent with earlier findings. Of the 6 anthocyanins found in *Vitis*, delphinidin is the least stable (He *et al.* 2010). Therefore, it is possible that the bronze genotypes had lower percent delphinidin composition than the black-fruited genotypes because the anthocyanins cannot be sequestered in the vacuole and this particular anthocyanin is degraded most rapidly in the cytosol. Although anthocyanin quantity varied significantly between black and bronze genotypes, the composition of the other 5 individual anthocyanins was similar among the 3 genotype classes (Fig. 6b).

## Conclusions

In this study, we isolated and characterized the candidate gene, *VrGST4*, responsible for berry color variation in muscadine grapes for the first time. We identified a nonsynonymous SNP (C/T) within *VrGST4* that corresponded to a proline to leucine mutation in bronze muscadines. A diagnostic KASP marker was developed from the intragenic SNP which cosegregated with berry color and allowed differentiation of bronze (TT), heterozygous black (CT), and homozygous black (CC) muscadines. *VrGST4* action was dominant; no allele dosage effects were observed on total anthocyanin content in black muscadines. *VrGST4* was highly expressed among in berry skins collected from black- and bronze-fruited genotypes at véraison. *VrGSTF12*, another Phi type GST located within the 0.8 Mbp berry color locus was excluded as a possible candidate for berry color in muscadine grapes because it was not expressed in berry skins from any genotypes. Taken together, our results suggest that berry color variation in muscadines is controlled by a mechanism different from that reported in *V. vinifera*, though the MYB genes that regulate the anthocyanin biosynthetic pathway in *Vitis* may have an important role in quantitative variation in anthocyanin content in dark-fruited muscadine grapes. These results will not only have important implications in muscadine breeding programs and the muscadine processing industry but also will provide insights in understanding the evolutionary pathways of *Vitis* species. Furthermore, this study sheds new light on the differential expression and regulation of transporter genes like GSTs in muscadine grapes and will provide new research avenues to elucidate the mechanisms involved in flavonoid pathway during fruit development and ripening.

## Data availability

Supplementary File 1 contains a list with detailed information for all the supplementary tables and figures. cDNA sequence data from this project has been deposited in NCBI GenBank with the following accession numbers; VrGST4-“Fry”: MT678460; VrGST4-“Supreme”: MT678462; VrGST4-“Black Beauty”: MT678461; VrGST4-“Nesbitt”: MT678463. RNA sequencing data generated in this project are deposited in NCBI under BioProject ID PRJNA784633. Other data generated and analyzed during this study are included in this published article and its supplementary information files.

Supplemental material is available at *G3* online.

## Supplementary Material

jkac060_Figure_S1

jkac060_Figure_S2

jkac060_Figure_S3

jkac060_Figure_S4

jkac060_Figure_S5

jkac060_Figure_S6

jkac060_Figure_S7

jkac060_Figure_S8

jkac060_File_S1

jkac060_Table_S1

jkac060_Table_S2

jkac060_Table_S3

jkac060_Table_S4
