# Supplementary material for: Glutathione S-transferase: a candidate gene for berry color in muscadine grapes (Vitis rotundifolia)
Source: G3 (Bethesda). 2022 Mar 18;12(5):jkac060. doi: 10.1093/g3journal/jkac060 (PMC9073687; doi:10.1093/g3journal/jkac060)
Supplement: jkac060_Figure_S1 [file jkac060_figure_s1.docx]

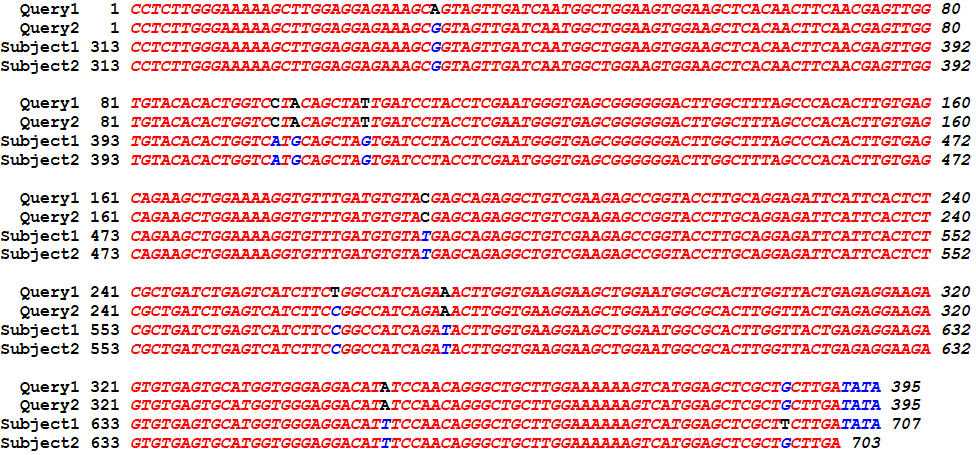


Figure S1. Sequence alignment of the 395 bp PCR product from genomic DNA of ‘Fry’ (Query1) and ‘Supreme’ (Query2) muscadines with *VaGST4* (Subject1) and *VvGST4* (Subject2) sequences from *Vitis amurensis* and *V. vinifera*, respectively. Numbers in the alignment represent base pairs.
