# Supplementary figures and images for: Glutathione S-transferase: a candidate gene for berry color in muscadine grapes (Vitis rotundifolia)
Source: G3 (Bethesda). 2022 Mar 18;12(5):jkac060. doi: 10.1093/g3journal/jkac060 (PMC9073687; doi:10.1093/g3journal/jkac060)

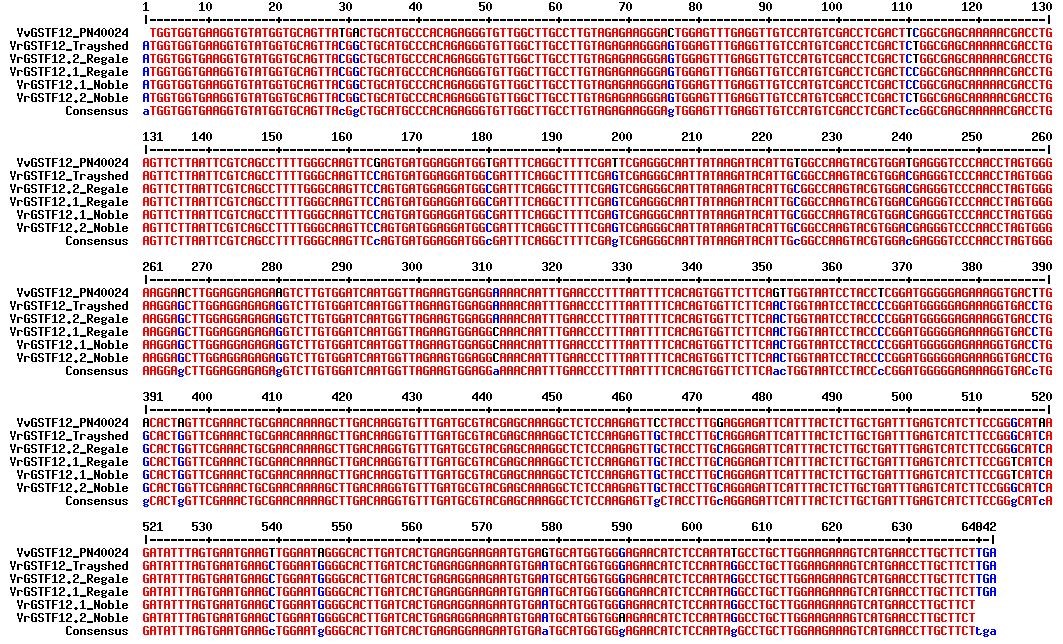

Supplement: jkac060_Figure_S2 [file jkac060_figure_s2.jpeg]

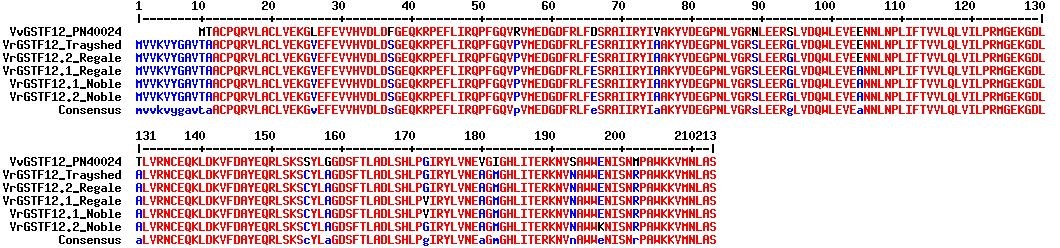

Supplement: jkac060_Figure_S3 [file jkac060_figure_s3.jpeg]

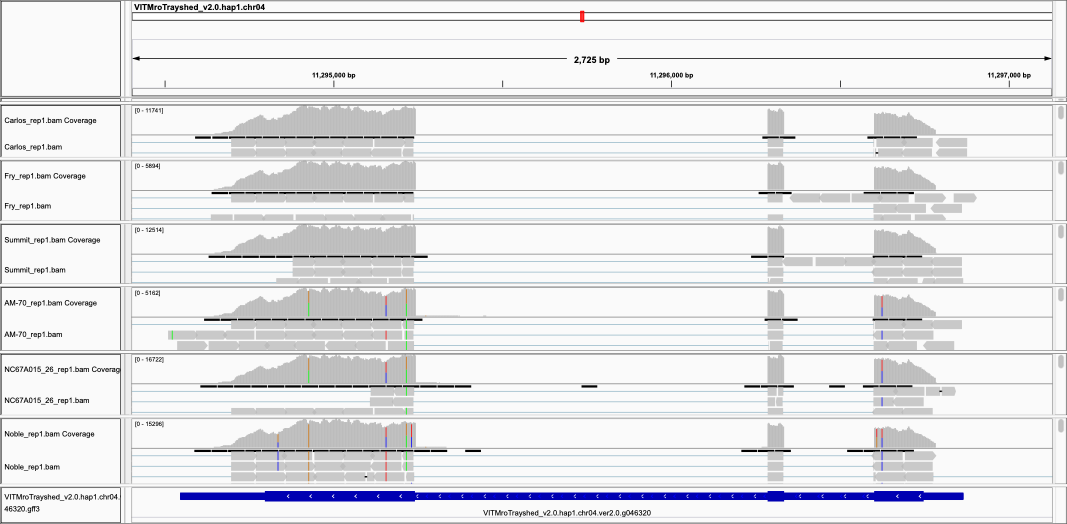

Supplement: jkac060_Figure_S4 [file jkac060_figure_s4.docx]

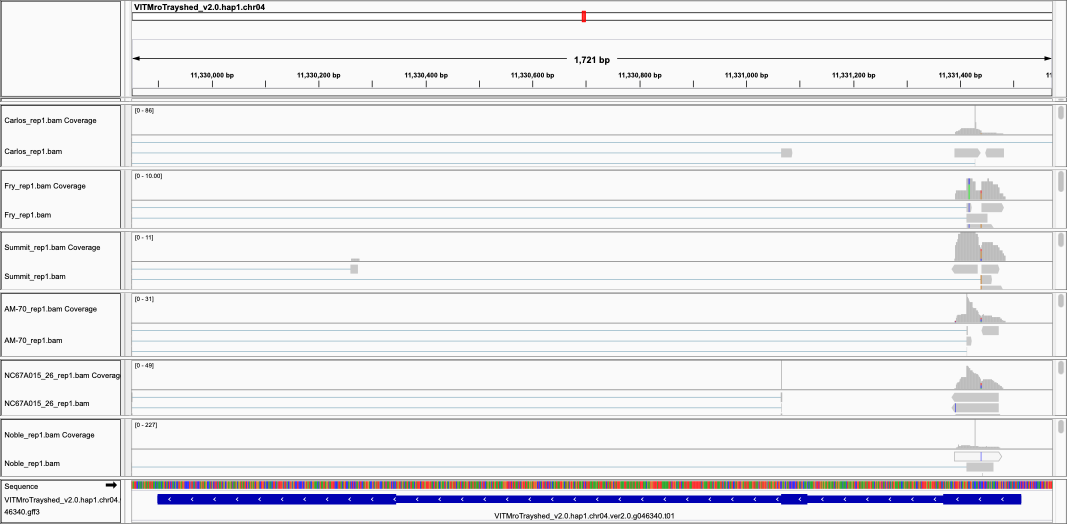

Supplement: jkac060_Figure_S5 [file jkac060_figure_s5.docx]

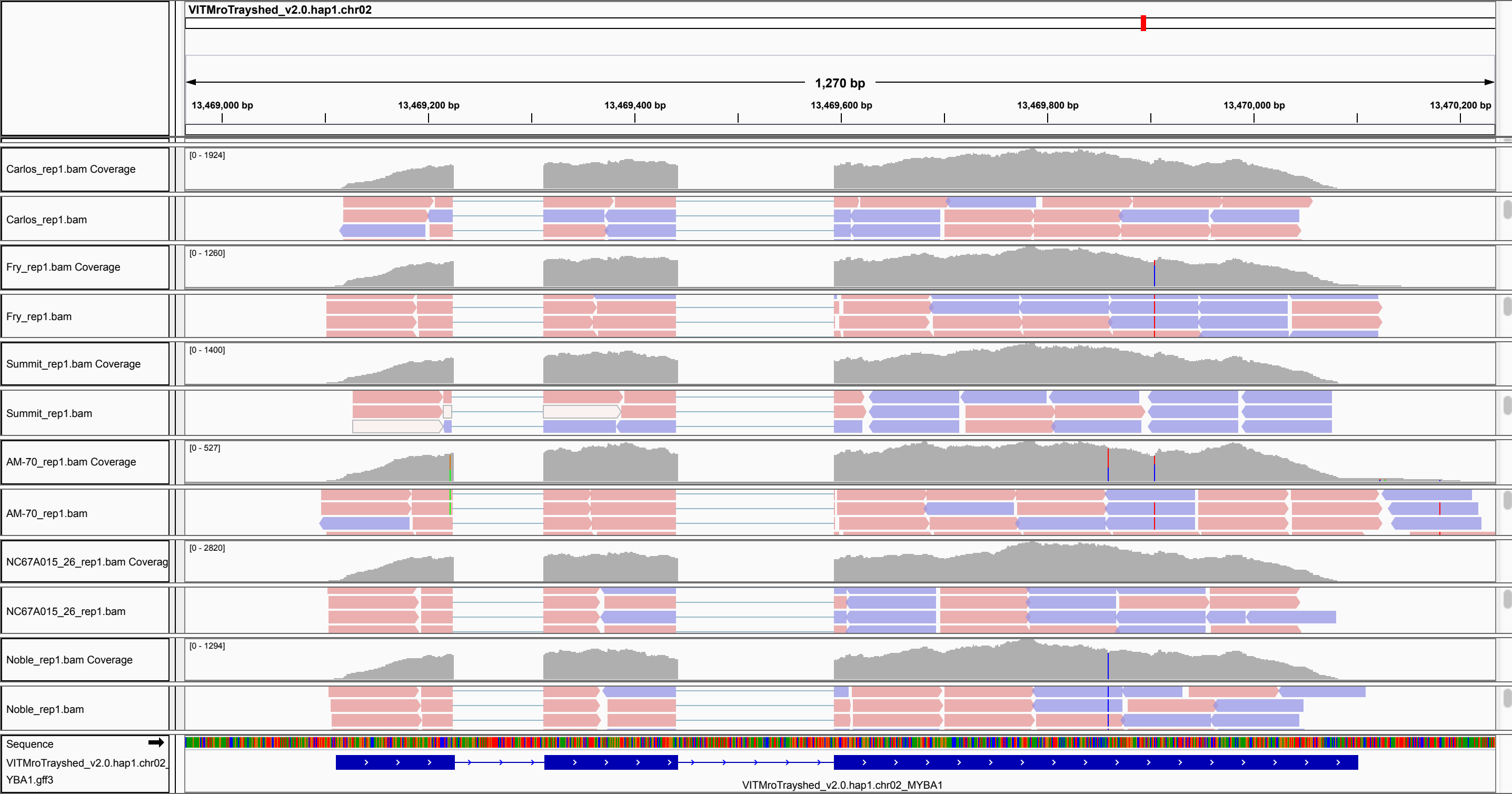

Supplement: jkac060_Figure_S6 [file jkac060_figure_s6.pdf]
