# Supplementary material for: Glutathione S-transferase: a candidate gene for berry color in muscadine grapes (Vitis rotundifolia)
Source: G3 (Bethesda). 2022 Mar 18;12(5):jkac060. doi: 10.1093/g3journal/jkac060 (PMC9073687; doi:10.1093/g3journal/jkac060)
Supplement: jkac060_Figure_S7 [file jkac060_figure_s7.docx]

PN40024 ATGGAGAGCTTAGGAGTTAGAAAGGGTGCATGGATCCAAGAAGAGGATGTTCTCCTGAGG 60

Trayshed ATGGAGGGCTTCGGAGTTAGAAAGGGTGCATGGACCCAAGAAGAGGATGTTCTCCTGAGG 60

Fry ATGGAGGGCTTCGGAGTTAGAAAGGGTGCATGGACCCAAGAAGAGGATGTTCTCCTGAGG 60

AM-70 ATGGAGGGCTTCGGAGTTAGAAAGGGTGCATGGACCCAAGAAGAGGATGTTCTCCTGAGG 60

Noble ATGGAGGGCTTCGGAGTTAGAAAGGGTGCATGGACCCAAGAAGAGGATGTTCTCCTGAGG 60

****** **** ********************** *************************

PN40024 AAATGCATTGAGAAATATGGAGAAGGAAAGTGGCATCTGGTTCCCCTCCGAGCAGGGTTG 120

Trayshed AAATGCATTGAGAAATATGGAGAAGGAAAGTGGCATCTGATTCCCCTCCGAGCAGGATTG 120

Fry AAATGCATTGAGAAATATGGAGAAGGAAAGTGGCATCTGATTCCCCTCCGAGCAGGATTG 120

AM-70 AAATGCATTGAGAAATATGGAGAAGGAAAGTGGCATCTGATTCCCCTCCCAGCAGGATTG 120

Noble AAATGCATTGAGAAATATGGAGAAGGAAAGTGGCATCTGATTCCCCTCCGAGCAGGATTG 120

*************************************** ********* ****** ***

PN40024 AATAGATGCCGAAAAAGCTGCAGGTTGAGATGGCTCAATTATTTGAAGCCGGATATCAAG 180

Trayshed AATAGATGCCGAAAAAGCTGCAGATTGAGATGGCTCAATTATTTGAAGCCGGATATCAAG 180

Fry AATAGATGCCGAAAAAGCTGCAGATTGAGATGGCTCAATTATTTGAAGCCGGATATCAAG 180

AM-70 AATAGATGCCGAAAAAGCTGCAGATTGAGATGGCTCAATTATTTGAAGCCGGATATCAAG 180

Noble AATAGATGCCGAAAAAGCTGCAGATTGAGATGGCTCAATTATTTGAAGCCGGATATCAAG 180

*********************** ************************************

PN40024 AGAGGAGAGTTTGCATTAGACGAGGTTGATCTCATGATTAGGCTTCACAATTTGTTGGGG 240

Trayshed AGAGGAGAGTTTGCATTAGACGAGGTTGATCTCATGATCAGGCTTCACAATTTGTTGGGG 240

Fry AGAGGAGAGTTTGCATTAGACGAGGTTGATCTCATGATCAGGCTTCACAATTTGTTGGGG 240

AM-70 AGAGGAGAGTTTGCATTAGACGAGGTTGATCTCATGATCAGGCTTCACAATTTGTTGGGG 240

Noble AGAGGAGAGTTTGCATTAGACGAGGTTGATCTCATGATCAGGCTTCACAATTTGTTGGGG 240

************************************** *********************

PN40024 AACAGATGGTCCTTGATTGCGGGTAGGCTTCCAGGGAGGACTGCTAATGATGTCAAGAAC 300

Trayshed AACAGATGGTCCTTGATTGCGGGTAGGCTTCCAGGGAGGACTGCTAATGATGTCAAGAAC 300

Fry AACAGATGGTCCTTGATTGCGGGTAGGCTTCCAGGGAGGACTGCTAATGATGTCAAGAAC 300

AM-70 AACAGATGGTCCTTGATTGCGGGTAGGCTTCCAGGGAGGACTGCTAATGATGTCAAGAAC 300

Noble AACAGATGGTCCTTGATTGCGGGTAGGCTTCCAGGGAGGACTGCTAATGATGTCAAGAAC 300

************************************************************

PN40024 TATTGGCATAGTCACCACTTCAAAAAGGAGGTTCAGTTCCAGGAAGAAGGGAGAGATAAA 360

Trayshed TATTGGCATAGTCACCACTTCAAAAAGAAGGTTCAGTTCCAGGAAGAAGGGAGAGAAAAA 360

Fry TATTGGCATAGTCACCACTTCAAAAAGAAGGTTCAGTTCCAGGAAGAAGGGAGAGAAAAA 360

AM-70 TATTGGCATAGTCACCACTTCAAAAAGAAGGTTCAGTTCCAGGAAGAAGGGAGAGAAAAA 360

Noble TATTGGCATAGTCACCACTTCAAAAAGAAGGTTCAGTTCCAGGAAGAAGGGAGAGAAAAA 360

*************************** **************************** ***

PN40024 CCCCAAACACATTCTAAAACCAAAGCTATAAAGCCTCACCCTCACAAGTTCTCCAAAGCC 420

Trayshed CCCCAAACACATTCTAAGACCAAAGCTATAAAGCCTCACCCTCACAAGTTCTCCAAAGCC 420

Fry CCCCAAACACATTCTAAGACCAAAGCTATAAAGCCTCACCCTCACAAGTTCTCCAAAGCC 420

AM-70 CCCCAAACACATTCTAAGACCAAAGCTATAAAGCCTCACCCTCACAAGTTCTCCAAAGCC 420

Noble CCCCAAACACATTCTAAGACCAAAGCTATAAAGCCTCACCCTCACAAGTTCTCCAAAGCC 420

***************** ******************************************

PN40024 TTGCCAAGGTTTGAACTAAAAACTACAGCTGTGGATACTTTTGACACACAAGTCAGTACT 480

Trayshed TTGCCAAGGTTTGAACTAAAAACTACAGCTGTGGATACTTTTGACACACAAGTAAGTACT 480

Fry TTGCCAAGGTTTGAACTAAAAACTACAGCTGTGGATACTTTTGACACACAAGTAAGTACT 480

AM-70 TTGCCAAGGTTTGAACTAAAAACTACAGCTGTGGATACTTTTGACACACAAGTAAGTACT 480

Noble TTGCCAAGGTTTGAACTAAAAACTACAGCTGTGGATACTTTTGACACACAAGTAAGTACT 480

***************************************************** ******

PN40024 TCCAGGAAGCCATCATCCACTTCACCACAACCGAATGATGACATCATATGGTGGGAAAGC 540

Trayshed TCCAGGAAGTCATCATCCACGTCACCACAACTGAATGATGACATTATATGGTGGGAAAGC 540

Fry TCCAGGAAGTCATCATCCACGTCACCACAACTGAATGATGACATTATATGGTGGGAAAGC 540

AM-70 TCCAGGAAGTCATCATCCACGTCACCACAACCGAATGATGACATTATATGGTGGGAAAGC 540

Noble TCCAGGAAGTCATCATCCACGTCACCACAACCGAATGATGACATTATATGGTGGGAAAGC 540

********* ********** ********** ************ ***************

PN40024 CTGTTAGCTGAGCATGCTCAAATGGATCAAGAAACTGACTTTTCGGCTTCTGGAGAGATG 600

Trayshed CTGTTAGCTGAGCATGCTCAAATGGATCAAGAAACTGACTTTTCGGCTTCTGTAGATGTG 600

Fry CTGTTAGCTGAGCATGTTCAAATGGATCAAGAAACTGACTTTTCGGCTTCTGTAGATGTG 600

AM-70 CTGTTAGCTGAGCATGTTCAAATGGATCAAGAAACTGACTTTTCGGCTTCTGTAGATGTG 600

Noble CTGTTAGCTGAGCATGCTCAAATGGATCAAGAAACTGACTTTTCGGCTTCTGTAGATGTG 600

**************** *********************************** *** **

PN40024 CTTATCGCAAGCCTCAGGACAGAAGAAACTGCAACACAGAAAAAGGGACCCATGGATGGT 660

Trayshed CTTATCGCAAGCCTCTGGACAGAAGAAACTGAAACACAGAAAAAGGTACCCATGGATTGT 660

Fry CTTATCGCAAGCCTCTGGACAGAAGAAACTGAAACACAGAAAAAGGTACCCATGGATTGT 660

AM-70 CTTATCGCAAGCCTCTGGACAGAAGAAACTGAAACACAGAAAAAGGTACCCATGGATTGT 660

Noble CTTATCGCAAGCCTCTGGACAGAAGAAACTGAAACACAGAAAAAGGTACCCATGGATTGT 660

*************** *************** ************** ********** **

PN40024 ATGATTGAACAAATCCAGGGAGGTGAGGGTGATTTTCCATTTGATGTGGGCTTCTGGGAT 720

Trayshed ATGACTGAACAAATCCAGGGAGGTGAGTGTGATTTTCCATTTGATGTGGGCTTCTGGGAT 720

Fry ATGACTGAACAAATCCAGGGAGGTGAGTGTGATTTTCCATTTGATGTGGGCTTCTGGGAT 720

AM-70 ATGACTGAACAAATCCAGGGAGGTGAGTGTGATTTTCCATTTGATGTGGGCTTCTGGGAT 720

Noble ATGACTGAACAAATCCAGGGAGGTGAGTGTGATTTTCCATTTGATGTGGGCTTCTGGGAT 720

**** ********************** ********************************

PN40024 ACACCCAACACACAAGTAAATCACTTGATCTGA 753

Trayshed ACACCCAACACGCAAATAAATCATTTGATCTGA 753

Fry ACACCCAACACGCAAATAAATCATTTGATCTGA 753

AM-70 ACACCCAACACGCAAATAAATCATTTGATCTGA 753

Noble ACACCCAACACGCAAATAAATCATTTGATCTGA 753

*********** *** ******* *********
