# Supplementary material for: Glutathione S-transferase: a candidate gene for berry color in muscadine grapes (Vitis rotundifolia)
Source: G3 (Bethesda). 2022 Mar 18;12(5):jkac060. doi: 10.1093/g3journal/jkac060 (PMC9073687; doi:10.1093/g3journal/jkac060)
Supplement: jkac060_Figure_S8 [file jkac060_figure_s8.docx]

PN40024 MESLGVRKGAWIQEEDVLLRKCIEKYGEGKWHLVPLRAGLNRCRKSCRLRWLNYLKPDIK 60

Trayshed MEGFGVRKGAWTQEEDVLLRKCIEKYGEGKWHLIPLRAGLNRCRKSCRLRWLNYLKPDIK 60

Fry MEGFGVRKGAWTQEEDVLLRKCIEKYGEGKWHLIPLRAGLNRCRKSCRLRWLNYLKPDIK 60

AM-70 MEGFGVRKGAWTQEEDVLLRKCIEKYGEGKWHLIPLRTGLNRCRKSCRLRWLNYLKPDIK 60

Noble MEGFGVRKGAWTQEEDVLLRKCIEKYGEGKWHLIPLRAGLNRCRKSCRLRWLNYLKPDIK 60

**.:******* *********************:***:**********************

PN40024 RGEFALDEVDLMIRLHNLLGNRWSLIAGRLPGRTANDVKNYWHSHHFKKEVQFQEEGRDK 120

Trayshed RGEFALDEVDLMIRLHNLLGNRWSLIAGRLPGRTANDVKNYWHSHHFKKKVQFQEEGREK 120

Fry RGEFALDEVDLMIRLHNLLGNRWSLIAGRLPGRTANDVKNYWHSHHFKKKVQFQEEGREK 120

AM-70 RGEFALDEVDLMIRLHNLLGNRWSLIAGRLPGRTANDVKNYWHSHHFKKKVQFQEEGREK 120

Noble RGEFALDEVDLMIRLHNLLGNRWSLIAGRLPGRTANDVKNYWHSHHFKKKVQFQEEGREK 120

*************************************************:********:*

PN40024 PQTHSKTKAIKPHPHKFSKALPRFELKTTAVDTFDTQVSTSRKPSSTSPQPNDDIIWWES 180

Trayshed PQTHSKTKAIKPHPHKFSKALPRFELKTTAVDTFDTQVSTSRKSSSTSPQLNDDIIWWES 180

Fry PQTHSKTKAIKPHPHKFSKALPRFELKTTAVDTFDTQVSTSRKSSSTSPQLNDDIIWWES 180

AM-70 PQTHSKTKAIKPHPHKFSKALPRFELKTTAVDTFDTQVSTSRKSSSTSPQPNDDIIWWES 180

Noble PQTHSKTKAIKPHPHKFSKALPRFELKTTAVDTFDTQVSTSRKSSSTSPQPNDDIIWWES 180

******************************************* ****** *********

PN40024 LLAEHAQMDQETDFSASGEMLIASLRTEETATQKKGPMDGMIEQIQGGEGDFPFDVGFWD 240

AM-70 LLAEHVQMDQETDFSASVDVLIASLWTEETETQKKVPMDCMTEQIQGGECDFPFDVGFWD 240

Noble LLAEHAQMDQETDFSASVDVLIASLWTEETETQKKVPMDCMTEQIQGGECDFPFDVGFWD 240

Trayshed LLAEHAQMDQETDFSASVDVLIASLWTEETETQKKVPMDCMTEQIQGGECDFPFDVGFWD 240

Fry LLAEHVQMDQETDFSASVDVLIASLWTEETETQKKVPMDCMTEQIQGGECDFPFDVGFWD 240

*****.*********** ::***** **** **** *** * ******* **********

PN40024 TPNTQVNHLI 250

AM-70 TPNTQINHLI 250

Noble TPNTQINHLI 250

Trayshed TPNTQINHLI 250

Fry TPNTQINHLI 250

*****:****
