# Supplementary material for: Glutathione S-transferase: a candidate gene for berry color in muscadine grapes (Vitis rotundifolia)
Source: G3 (Bethesda). 2022 Mar 18;12(5):jkac060. doi: 10.1093/g3journal/jkac060 (PMC9073687; doi:10.1093/g3journal/jkac060)
Supplement: jkac060_File_S1 [file jkac060_file_s1.docx]

**File S1. List of Supplemental Figures and Tables**

***Supplemental figures***

**Figure S1** Sequence alignment of the 395 bp PCR product from genomic DNA of ‘Fry’ (Query1) and ‘Supreme’ (Query2) muscadines with *VaGST4* (Subject1) and *VvGST4* (Subject2) sequences from *Vitis amurensis* and *V. vinifera*, respectively. Numbers in the alignment represent base pairs.

**Figure S2** Alignment of *GSTF12* coding sequences from *V. vinifera* PN40024 and ‘Trayshed’ (VITMroTrayshed_v1.1_EVM.ver0.0.g1.04.943.1.t01), ‘Noble’, and ‘Regale’ muscadine cultivars. Numbers in the alignment represent base pairs. PN40024, ‘Regale’, and ‘Noble’ are black-fruited, while ‘Trayshed’ is a bronze genotype.

**Figure S3** Alignment of predicted GSTF12 proteins from *V. vinifera* PN40024 and ‘Trayshed’ (VITMroTrayshed_v1.1_EVM.ver0.0.g1.04.943.1.t01), ‘Noble’, and ‘Regale’ muscadine cultivars. Numbers in the alignment represent amino acid positions. PN40024, ‘Regale’, and ‘Noble’ are black-fruited, while ‘Trayshed’ is a bronze genotype.

**Figure S4** Alignments of the RNA-seq read from berries of three bronze and three black genotypes on the ‘Trayshed’ muscadine genome at the *VrGST4* gene locus (VITMroTrayshed_v2.0.hap1.chr04.ver2.0.g046320). The three bronze genotypes are ‘Carlos, Fry’, and ‘Summit’. The three black genotypes are AM-70, NC67A015_26, and ‘Noble’. Only the first biological replicate of each genotype is represented.

**Figure S5** Alignments of the RNA-seq read from berries of three bronze and three black genotypes on the ‘Trayshed’ muscadine genome at the *VrGSTF12* gene locus (VITMroTrayshed_v2.0.hap1.chr04.ver2.0.g046340). The three bronze genotypes are ‘Carlos’, ‘Fry’, and ‘Summit’. The three black genotypes are AM-70, NC67A015_26, and ‘Noble’. Only the first biological replicate of each genotype is represented.

**Figure S6** Alignments of the RNA-seq read from berries of three bronze and three black genotypes on the ‘Trayshed’ muscadine genome at the *VrMybA1* gene locus (VITMroTrayshed_v2.0.hap1.chr02.ver2.0.g024710). The three bronze genotypes are ‘Carlos’, ‘Fry’, and ‘Summit’. The three black genotypes are AM-70, NC67A015_26, and ‘Noble’. Only the first biological replicate of each genotype is represented.

**Figure S7** Alignment of *MybA1* coding sequences from *V. vinifera* PN40024 (VviMYBA1_VIT_02s0033g00410.t01) and ‘Trayshed’ (VITMroTrayshed_v2.0.hap1.chr02.ver2.0.g024710) assemblies with ‘Fry’, ‘AM-70’, and ‘Noble’ sequences deduced from RNA sequencing. The NC67A015_26, ‘Carlos’, and ‘Summit’ coding sequences were all identical to ‘Trayshed’. Numbers in the alignment represent base pairs. Nonsynonymous polymorphisms within the four *V. rotundifolia* genotypes are highlighted in yellow. Single nucleotide polymorphisms (SNPs) in the heterozygous condition are colored red and homozygous SNPs are colored green. PN40024, ‘AM-70’, and ‘Noble’ are black-fruited, while ‘Trayshed’ and ‘Fry’ are bronze genotypes.

**Figure S8** Alignment of predictied MybA1 proteins from *V. vinifera* PN40024 (VviMYBA1_VIT_02s0033g00410.t01) and ‘Trayshed’ (VITMroTrayshed_v2.0.hap1.chr02.ver2.0.g024710) assemblies with ‘Fry’, ‘AM-70’, and ‘Noble’ sequences deduced from RNA sequencing. Predicted protein sequences for NC67A015_26, ‘Carlos’, and ‘Summit’ were all identical to ‘Trayshed’. Numbers in the alignment represent amino acid positions. Amino acid substitutions within the four *V. rotundifolia* genotypes are highlighted in yellow. Substitutions in the heterozygous condition are colored red and homozygous substitutions are colored green. PN40024, ‘AM-70’, NC67A015_26, and ‘Noble’ are black-fruited, while ‘Trayshed’, ‘Carlos’, ‘Fry’, and ‘Summit’ are bronze genotypes.

***Supplemental tables***

**Table S1** BLASTn results from the sequence alignment of the 395 bp PCR product from genomic DNA of ‘Fry’ (Query1) and ‘Supreme’ (Query2) muscadines with VaGST4 (Subject1) and VvGST4 (Subject2) sequences from *Vitis amurensis* and *V. vinifera*, respectively.

**Table S2** Summary statistics of RNA sequencing.

**Table S3** BLASTn results from the sequence alignment of the PCR product amplified from synthetic plasmids of cDNA sequences of *VrGSTF12* from ‘Trayshed’ (Query 1 and Query 4) and ‘Noble’ (Query 2, Query 3, and Query 5) with the CDS sequence of *VrGSTf12* (VITMroTrayshed_v1.1_EVM.ver0.0.g1.04.943.1.t01) in the ‘Trayshed’ assembly (Subject 1). Queries 1-3 are products amplified using forward primer 5’ AATGGAAGATGGTGGTGAAG 3’ and reverse primer 5’ GGATCTCAAGAAGCAAGGTT 3’, while Queries 4 and 5 were amplified with forward primer 5’ ATGGTGGTGAAGGTGTATGGTG 3’ and reverse primer 5’ TCAAGAAGCAAGGTTCATGACTTTC 3’.

**Table S4** Berry color phenotypes and KASP predicted *VrGST4* genotypes of 76 cultivars, 126 breeding selections, and 359 progeny from three muscadine mapping populations.
