# Supplementary material for: Glutathione S-transferase: a candidate gene for berry color in muscadine grapes (Vitis rotundifolia)
Source: G3 (Bethesda). 2022 Mar 18;12(5):jkac060. doi: 10.1093/g3journal/jkac060 (PMC9073687; doi:10.1093/g3journal/jkac060)
Supplement: jkac060_Table_S1 [file jkac060_table_s1.docx]

Table S1: BLASTn results from the sequence alignment of the 395 bp PCR product from genomic DNA of ‘Fry’ (Query1) and ‘Supreme’ (Query2) muscadines with *VaGST4* (Subject1) and VvGST4 (Subject2) sequences from *Vitis amurensis* and *V. vinifera*, respectively.

| Alignment | Identities (%) | Gaps (%) | Expect Value | Score (bits) |
| --- | --- | --- | --- | --- |
| Query1/Subject1 | 97.72 | 0 | 0.0 | 680 |
| Query1/Subject2 | 97.95 | 0 | 0.0 | 678 |
| Query2/Subject1 | 98.23 | 0 | 0.0 | 691 |
| Query2/Subject2 | 98.47 | 0 | 0.0 | 689 |
