# Supplementary material for: Glutathione S-transferase: a candidate gene for berry color in muscadine grapes (Vitis rotundifolia)
Source: G3 (Bethesda). 2022 Mar 18;12(5):jkac060. doi: 10.1093/g3journal/jkac060 (PMC9073687; doi:10.1093/g3journal/jkac060)
Supplement: jkac060_Table_S3 [file jkac060_table_s3.docx]

Table S2: BLASTn results from the sequence alignment of the PCR product amplified from synthetic plasmids of cDNA sequences of *VrGSTF12* from ‘Trayshed’ (Query1 and Query4) and ‘Noble’ (Query2, Query3, and Query 5) with the CDS sequence of *VrGSTF12* (VITMroTrayshed_v1.1_EVM.ver0.0.g1.04.943.1.t01) in the ‘Trayshed’ assembly (Subject 1). Queries 1-3 are products amplified using forward primer 5’ AATGGAAGATGGTGGTGAAG 3’ and reverse primer 5’ GGATCTCAAGAAGCAAGGTT 3’, while Queries 4 and 5 were amplified with forward primer 5’ ATGGTGGTGAAGGTGTATGGTG 3’ and reverse primer 5’ TCAAGAAGCAAGGTTCATGACTTTC 3’.

| Alignment | Identities (%) | Gaps (%) | Expect Value | Score (bits) |
| --- | --- | --- | --- | --- |
| Query1/Subject1 | 99.31 | 0.00 | 0.0 | 1142 |
| Query2/Subject1 | 98.77 | 0.18 | 0.0 | 1104 |
| Query3/Subject1 | 99.83 | 0.00 | 0.0 | 1206 |
| Query4/Subject1 | 99.64 | 0.00 | 0.0 | 1098 |
| Query5/Subject1 | 99.46 | 0.00 | 0.0 | 1094 |
